# Community-based care models for arterial hypertension management in non-pregnant adults in sub-Saharan Africa: a literature scoping review and framework for designing chronic services

**DOI:** 10.1186/s12889-022-13467-4

**Published:** 2022-06-04

**Authors:** Lucia González Fernández, Emmanuel Firima, Elena Robinson, Fabiola Ursprung, Jacqueline Huber, Alain Amstutz, Ravi Gupta, Felix Gerber, Joalane Mokhohlane, Thabo Lejone, Irene Ayakaka, Hongyi Xu, Niklaus Daniel Labhardt

**Affiliations:** 1 Department of Medicine, Swiss Tropical and Public Health Institute, 4051 Allschwil, Basel, Switzerland; 2 University of Basel, 4001 Basel, Switzerland; 3 SolidarMed, Partnerships for Health, Maseru, 0254 Lesotho; 4 Faculty of Biomedical Sciences, Università Della Svizzera Italiana, 6900 Lugano, Switzerland; 5 Swiss TPH Library, Swiss Tropical and Public Health Institute, 4051 Allschwil, Basel, Switzerland; 6 Non-Communicable Diseases Unit, Ministry of Health, Maseru, 174 Lesotho; 7 Department of Non-Communicable Diseases, World Health Organization, 1202 Geneva, Switzerland; 8 Department of Infectious Diseases and Hospital Epidemiology, University Hospital Basel, 4031 Basel, Switzerland

**Keywords:** Arterial hypertension, Hypertension treatment, Cardiovascular disease, Implementation research, Community-based care, Out-of-facility care, Non-communicable diseases, Chronic diseases, Chronic care services, Models of care, Health systems, Sub-Saharan Africa

## Abstract

**Background:**

Arterial hypertension (aHT) is the leading cardiovascular disease (CVD) risk factor in sub-Saharan Africa; it remains, however, underdiagnosed, and undertreated. Community-based care services could potentially expand access to aHT diagnosis and treatment in underserved communities. In this scoping review, we catalogued, described, and appraised community-based care models for aHT in sub-Saharan Africa, considering their acceptability, engagement in care and clinical outcomes. Additionally, we developed a framework to design and describe service delivery models for long-term aHT care.

**Methods:**

We searched relevant references in Embase Elsevier, MEDLINE Ovid, CINAHL EBSCOhost and Scopus. Included studies described models where substantial care occurred outside a formal health facility and reported on acceptability, blood pressure (BP) control, engagement in care, or end-organ damage. We summarized the interventions’ characteristics, effectiveness, and evaluated the quality of included studies. Considering the common integrating elements of aHT care services, we conceptualized a general framework to guide the design of service models for aHT.

**Results:**

We identified 18,695 records, screened 4,954 and included twelve studies. Four types of aHT care models were identified: services provided at community pharmacies, out-of-facility, household services, and aHT treatment groups. Two studies reported on acceptability, eleven on BP control, ten on engagement in care and one on end-organ damage. Most studies reported significant reductions in BP values and improved access to comprehensive CVDs services through task-sharing. Major reported shortcomings included high attrition rates and their nature as parallel, non-integrated models of care. The overall quality of the studies was low, with high risk of bias, and most of the studies did not include comparisons with routine facility-based care.

**Conclusions:**

The overall quality of available evidence on community-based aHT care is low. Published models of care are very heterogeneous and available evidence is insufficient to recommend or refute further scale up in sub-Sahara Africa. We propose that future projects and studies implementing and assessing community-based models for aHT care are designed and described according to six building blocks: providers, target groups, components, location, time of service delivery, and their use of information systems.

**Supplementary Information:**

The online version contains supplementary material available at 10.1186/s12889-022-13467-4.

## Background and aim

The World Health Organization (WHO) defines aHT as a persistent systolic blood pressure (SBP) ≥ 140 mmHg or diastolic BP (DBP) ≥ 90 mmHg. An estimated 1.28 billion adults aged 30–79 years worldwide have aHT, two-thirds of them living in low- and middle-income countries. Modifiable risk factors include unhealthy diets (excessive salt consumption, saturated fat and trans fats, low intake of fruits and vegetables), physical inactivity, consumption of tobacco and alcohol, and overweight or obesity. Non-modifiable risk factors include a family history of aHT, age over 65 years, and co-existing diseases such as diabetes or kidney disease. aHT is the largest modifiable cardiovascular risk factor (CVRF) globally and the leading cause of the 22.9 million deaths attributed to cardiovascular diseases (CVDs) each year in sub-Saharan Africa [1–3].

Prevalence of aHT is highest in the African region, where an estimated 27% of the population aged 30–79 years have aHT [4]. However, despite an increasing burden of CVDs, aHT awareness, diagnosis, treatment and control remain low [5–8]. Barriers to aHT control exist at patient and health system levels [9, 10]. Major challenges for people living with aHT relate to the asymptomatic nature of the condition, leading to a delayed diagnosis and treatment initiation. Once diagnosed, aHT requires lifelong lifestyle modifications, frequent medical check-ups, ongoing counselling and regular adaptation of treatment dosage or drug regimen [9, 11]. Regional health systems remain poorly adapted to provide comprehensive CVD care, with insufficiently trained, equipped and supported workforce, limited availability of treatment options, and infrequent or non-existing monitoring of treatment outcomes, such as BP control and end-organ function [8, 12].

As aHT is a prevalent, chronic and, often, asymptomatic health condition, successful care models must be easy to access and provide long-term medical follow-up [13–16]. Community-based health services have been proposed as solutions to bridge existing barriers in access and to scale up services for aHT [17–19]. These care models frequently promote task-shifting/sharing, simplification of clinical care algorithms and integration of other services [20–25]. Although the terms task shifting and task sharing are sometimes used interchangeably: task shifting is defined as a systematic and planned transfer of care duties from physicians to non-physicians, such as nurses, or community health workers [26], whereas task sharing describes professionals working together to deliver health services. In practice, this implies that when physicians are not available, care tasks must be shifted to non-physician workers for the health system to function. When a few physicians are available, tasks may be shared with other health-care professionals with some supervision or referral to physicians [27, 28].

To date, community-based and out-of-facility care models have been applied to scale up treatment for HIV and tuberculosis (TB), with different success [29–35]. However, currently, there is no consensus nor guidance on how such models should be structured to have substantial impact in aHT care. Similarly, evidence to understand how, and to what extent tasks could be shifted to lower cadre health care providers, and how services could be decentralized is lacking. A preliminary search of MEDLINE, the Cochrane Database of Systematic Reviews and the Joanna Briggs Institute (JBI) Evidence Synthesis revealed no systematic reviews or similar scoping reviews on this topic. To inform future research, public health programs, and policies, this literature scoping review aims to catalogue the existing community-based aHT care models for non-pregnant adults in sub-Sahara Africa.

## Methods

We chose a scoping review methodology to provide an overview and a categorization of existing knowledge, rather than a narrow synthesis of a predefined research question. Typically, scoping reviews are used to map the key concepts that underpin a field of research, as well as to clarify working definitions, and/or the conceptual boundaries of a topic. In this scoping review, the authors explore the breadth of the literature, map and summarize the evidence, and inform future research in the topic [36]. We followed the framework proposed by Arksey and O’Malley [37], further developed by Levac et al. [38] and the Joanna Briggs Institute [39]. The protocol has been published [40]. Our primary objective is to construct a framework to categorize these aHT care models. Secondary objectives include: 1) to appraise the models of care, in terms of acceptability, BP control, engagement in care, and occurrence of end-organ damage, 2) to describe within-study comparisons between community-based and facility-based models of care, if provided by authors and 3) to identify gaps in the literature with respect to community-based service models for aHT.

We included studies in which participants were non-pregnant adults ≥ 18 years, diagnosed with aHT and living in sub-Saharan Africa. A summary of eligibility criteria is available in Table 1. Included studies had to report medical management and treatment for aHT that differed from conventional facility-based care in terms of provider cadre, location, or frequency of follow-up visits. Interventions had to address general management and medical treatment for aHT, including lifestyle modification, self-care, treatment administration and screening or management of organ complications. Included studies had also to report on at least one of the following outcomes: acceptability of the care model, BP control, engagement in care, or end-organ damage. We did not include studies where only aHT screening or diagnosis was reported. Studies where the intervention was a mere add-on and did not replace, at least partly, facility-based care, or did not reduce the frequency of visits to a professional health care worker, were not eligible. We also excluded studies that reported the pilot experience of a published intervention, if the model of care was the same.

**Table 1:** Study inclusion and exclusion criteria

**FIELD**	**INCLUSION CRITERIA**	**EXCLUSION CRITERIA**
POPULATION	Non-pregnant adults ≥ 18 years diagnosed for aHT Any gender	
GEOGRAPHIC REGION	sub-Saharan Africa, which includes the following countries: Angola, Benin, Botswana, Burkina Faso, Burundi, Cameroon, Central African Republic, Chad, Congo, Cote d'Ivoire, Equatorial New Guinea, Eritrea, Ethiopia, eSwatini, Gabon, Gambia, Ghana, Guinea, Guinea-Bissau, Kenya, Lesotho, Liberia, Madagascar, Malawi, Mali, Mauritania, Mauritius, Mozambique, Namibia, Niger, Nigeria, Rwanda, Senegal, Sierra Leone, Seychelles, Somalia, South Africa, Sudan (North, South), United Republic of Tanzania, Togo, Uganda, Zaire, Zambia, Zimbabwe	Studies conducted outside the sub-Saharan region
INTERVENTION/MODEL OF CARE AND OUTCOMES	Medical management and treatment for aHT, including health promotion strategies, self-care, and screening of complications, that differs from standard, facility-based or conventional care in terms of provider cadre, location, or frequency Studies that report at least one of the following outcomes: •Acceptability •Blood pressure control •Engagement in care •End-organ damage	Report solely about standard or conventional, facility-based model for delivering treatment Description does not describe the main characteristics needed to define the model Unable to provide sufficient description of at least one outcome of interest
SECTOR	Services provided in the public sector through government-managed public health infrastructure or through private or non-governmental programs or facilities that serve the uninsured sector	Services or programs for privately (commercially) insured patients
TYPE OF STUDIES	Peer-reviewed studies that provide the necessary data to assess at least one of the outcomes of interest, including prospective cohort studies, case control studies, randomized controlled trials, letters to editors, and qualitative studies on the topic	Treatment guidelines, mathematical models, conference abstracts that have not resulted in a peer-reviewed publication, editorials, viewpoints, commentaries. case reports, and systematic reviews
LANGUAGE	No limits	None
STUDY DATE	No limits	None

A first literature search was conducted on 23 May 2021. The search was repeated on 15 October 2021, yielding no further eligible studies. The literature search strategy was drafted and refined through discussions with the study team and an experienced scientific information specialist (JH), then reviewed by a second information specialist (CA). The search strategy was first developed in Embase Elsevier [41], and subsequently translated for the databases Medline Ovid [42], Cumulative Index to Nursing and Allied Health Literature (CINAHL) [43], and Scopus [44]. Detailed information on the search strategy and is available in Annex 1.

No language limits or date restrictions were applied. Conference abstracts where no peer-reviewed publication was available were excluded. The search results from each database were imported to EndNote X9 and deduplicated according to the method of Bramer [45]. Two independent reviewers (LG, ER) individually assessed study titles, abstracts, and full texts against the predefined eligibility criteria of the review. Authors were contacted when the description of the model of care was unclear or incomplete to decide on inclusion or exclusion. Backward and forward citation from included studies was used to identify additional articles that met the inclusion criteria. Disagreements were resolved through discussions between the two first authors (LGF, EF) and the last author (NDL).

Data extraction was independently conducted by two authors (LG, ER) using a tool created in Word™v.16.0 and piloted on three studies. Information included author, year of publication, study design, target population, location of study, duration of follow-up, type of community-care model, health provider cadre, outcomes measured and comparison arm if available. All reported variables were described as the authors defined them, with no other assumptions. Discrepancies between reviewers were discussed and solved by consensus. The information was chartered in Excel™ v16.0.

We summarized each study’s outcomes and, where possible, we pooled outcomes and reported average, range and/or median values. If models of care were similar, we grouped results by intervention type and reported common features, such as health cadre providing the service, location of delivery and frequency, use of e-Health, or integration with other chronic conditions. Assessment of quality of the included evidence was not initially planned, and thus not specified in the published protocol. However, we undertook the analysis at a later stage to comment on recommendations for evidence generation in the field. We assessed the quality of the included cohort and case–control studies using the Newcastle–Ottawa Scale [46]. The domains of the tool rate the selection of participants, comparability, and outcomes, to a maximum of 9 points. Whereas this scale is widely used to assess the quality of observational studies, there are no established thresholds to define “poor” or “good" quality of a study. Based on a recent literature review, we applied a threshold ≥ 6 as “no high risk of bias” [47]. Randomized controlled trials (RCTs) were judged using the Cochrane Collaboration’s tool to assess RCTs [48] and cluster RCTs [49]. We evaluated the sequence generation, participant recruitment with respect to randomization timing, deviation from intended intervention, completeness of outcome data for the main outcome, bias in the measurement of outcome, and bias in the selection of the reported result. Additionally, we addressed both quantitative and qualitative gaps in the literature and proposed suggestions for further studies and applications for programmatic scale up. The results of the review are documented in accordance with the PRISMA-P reporting checklist [50].

Using the reported experiences, we conceptualized a framework containing six building blocks to design and describe community care models for aHT.

## Results

### Search results

**Fig. 1:**
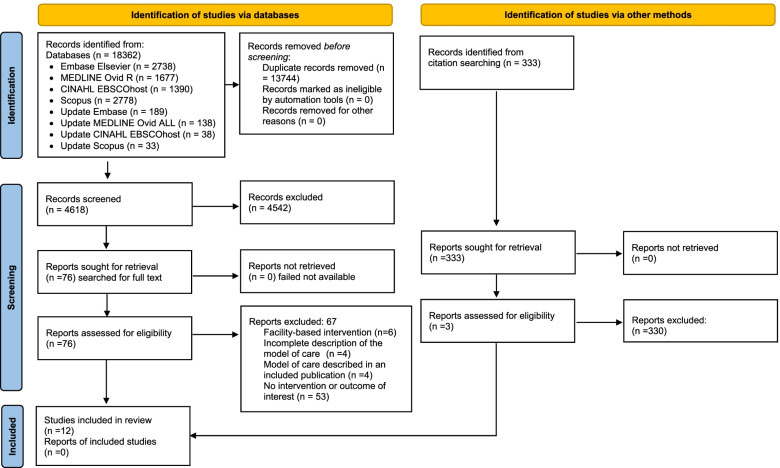
Studies selection PRISMA diagram. *From:* Page MJ, McKenzie JE, Bossuyt PM, Boutron I, Hoffmann TC, Mulrow CD, et al. The PRISMA 2020 statement: an updated guideline for reporting systematic reviews. BMJ 2021;372:n71. https://doi.org/10.1136/bmj.n71. https://doi.org/10.1136/bmj.n71, For more information, visit: http://www.prisma-statement.org/

Literature search and deduplication yielded a total of 4,618 citations (Prisma Fig. 1). Titles and abstracts screening resulted in a first classification, after which 76 papers were included for full-text review. Reasons for exclusion at full-text screening included: studies described models with most of the aHT care happening at facility level (*n* = 6); the description of the model of care lacked details on the content of the intervention (*n* = 4); studies piloted a model of care that was further described in an included study (*n* = 4); and the described model of care or outcomes did not match the inclusion criteria (*n* = 53). Backward and forward citation searching of included studies yielded 333 additional references; all of them were screened, and three new studies were identified. As a result, 12 references were finally included [51–62].

### Characteristics of the studies

**Fig. 2:**
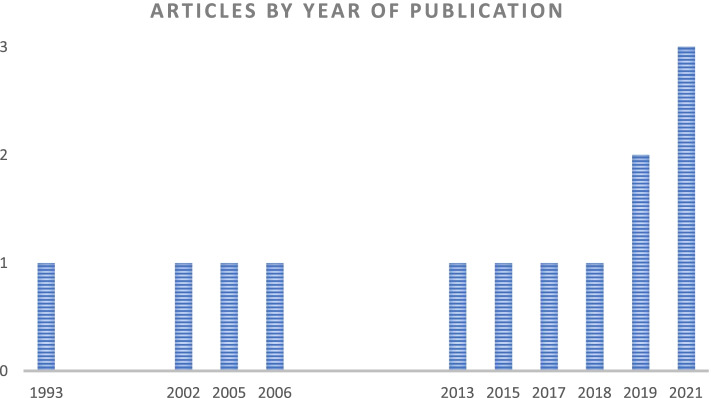
Included studies by year of publication

**Fig. 3:**
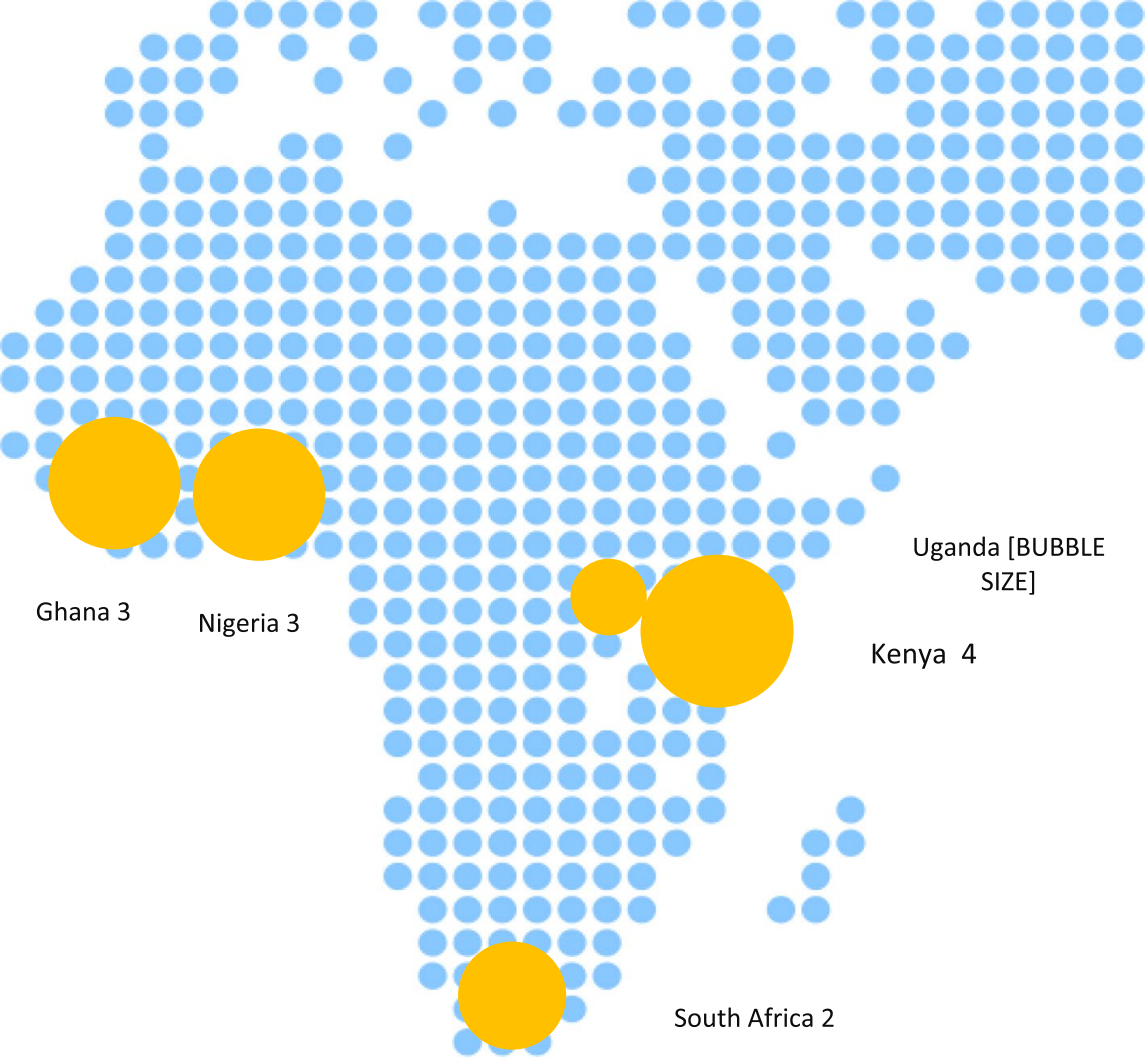
Included studies by country

Characteristics of included studies are available in Table 2. Identified studies were published between 1994 and 2021, and seven (58%) were published after 2017 [52–57, 59, 61] (Fig. 2). Eleven (92%) were single country studies [51–56, 58–62] whereas one [57] implemented the same service model in two countries. West African populations were represented in four (33%) [52, 55, 59, 60], East African populations in five (42%) [53, 54, 56, 58, 61], Southern African populations in two (17%) [51, 62] studies and one (8%) study presented results from both East and West Africa [57] (Fig. 3). Seven (58%) studies were conducted in urban areas [51, 54, 55, 58–61], three (25%) in rural areas [53, 56, 62], one (8%) study [52] took place in semi-urban areas and one (8%) [57] study reported findings in urban and rural settings. No studies reported interventions in special settings, such as remote, hard-to-reach populations or conflict areas.

**Table 2:** Baseline characteristics of studies

	**Author and publication year**	**Study design**	**Country and setting**	**Name project/model of care**	**Participants (eligibility criteria)**	**Sample size (*n*)**	**Study period**	**Comparisons**		**Integration with other services**	**Use of eHealth technology**
								**Intervention**	**Intervention**		
**1**	Steyn et al. 1993 [62]	Prospective quasi-experimental study with cohort and cross-sectional elements	South Arica semiurban (defined by authors as rural) towns	CORIS	Hypertensive patients, 15-64y at baseline, 15-68y at endline	7188	1979–1983	•1 low intensity intervention town: use of small mass media (billboards, posters, pamphlets) to deliver messages in the community •1 high intensity intervention town: hypertensives, active follow up through community BP stations and exposure to media messages	•Control town	General counselling on lifestyle related to CVDRFs	None
**2**	Oparah et al. 2006 [60]	Prospective cohort study	Nigeria urban	-	Hypertensive patients ≥ 18y on aHT treatment	42	2003–2004	•1 community pharmacy: pharmacists provided BP monitoring, BMI measurement, medication education, lifestyle modifications, and assistance with treatment compliance	•N/A	No	Follow up through phone calls
**3**	Ndou et al. 2013 [51]	Retrospective case control study	South Africa urban	Kgatelopele programme	Stable patients with hypertension or diabetes Three-fold matched controls	224	NR	•Monthly home visits by one CHWs. Pharmacist pre-packed a month’s supply of medication for delivery. Patients visit the clinic every 6 months for a physical examination by a doctor who provides a renewed prescription	•Clinic-based standard of care	Diabetes	None
**4**	Khabala et al. 2015 [58]	Retrospective cohort study	Kenya urban	Medication Adherence Clubs for multiple chronic diseases	Stable patients with diabetes, hypertension and/or HIV	1432	2013–2014	•MACs are nurse-facilitated, mixed groups of 25–35 stable hypertension, diabetes and/or HIV patients •Nurses lead quarterly meetings in medication adherence clubs (MACs) in health facilities to confirm clinical stability, have brief health discussions and receive medication •Clinical officer reviewed MACs yearly when patients developed complications or no longer met stability criteria	•N/A	Diabetes and HIV	None
**5**	Marfo et al. 2017 [55]	Prospective non-randomized controlled trial	Ghana urban	-	Patients diagnosed with hypertension ≥ 6 months, with a review period of at least two months	180	NR	•Monthly follow up at 3 community pharmacies: BP monitoring, medicines use review, health education and adherence counselling •Follow up reminders via text messages and phone calls	•2 control community pharmacies	Diabetes	Follow up through SMS and phone calls
**6**	Nelissen et al. 2018 [61]	Prospective mixed-methods study	Nigeria urban	-	Hypertensive patients > 18y. SBP ≤ 180 mmHg and DBP ≤ 110 mmHg. No history of cardiac failure, stroke, or renal disease. No additional CVRF. Non-pregnant	336	2016–2017	•5 community pharmacies where staff and cardiologists provide joint care directly connected through a mobile application (mHealth) for remote patient monitoring •Task-shifting from medical doctors to pharmacy staff: pharmacy staff performed regular follow up, including BP measurements, medication and lifestyle counselling, visits reminders and communication with the cardiologist	•N/A	None	Patients, pharmacists, and cardiologists connected through a mobile application: mHealth
**7**	Kuria et al. 2018 [54]	Retrospective cohort study	Kenya urban	-	Hypertensive patients retained in clinics for at least 6 months	785	2015–2016	•Model of care adapted to give services to a transient community •Weekend clinics in churches offered comprehensive services between 0900 and 1600 h, on worship days • “Walkways”, drop-in-clinics offered comprehensive care, located on commonly used roadways outside or near the clinic operating between 1630 and 1830 h •CHVs take BP readings. A clinician supervises the CHVs, diagnoses, treats patients, and dispenses medication. Clinicians are drawn from project sites and work on a rotational basis •A patient booklet containing clinical information is issued to address patients mobility	•Regular services at health facilities	None	None
**8**	Adler et al. 2019 [52]	Prospective cohort study	Ghana semiurban/peri-urban	ComHIP study	Diagnosed hypertension in ≥ 18y, non-pregnant, with access to a mobile phone	1339	2015–2016	•Monthly BP monitoring appointments, review visits every 1,2 or 3 months depending on risk and personal factors •6-monthly follow up assessments at local drug shops or CHWs •Daily adherence reminders and weekly healthy living tips by SMS	•N/A	None	-Electronic database CommCare -Cloud-based health records system that links patients’ records with SMS system -SMS platform automatically sends daily adherence reminders, weekly healthy living tips, and consultation and prescription refill reminders to enrolled patients
**9**	Bolarinwa et al. 2019 [59]	Unblinded individual open RCT	Nigeria urban	-	Hypertensive adults on treatment	299	NR	•Monthly follow up visits at home conducted by nurses including counselling, education, family approaches and integration of other chronic conditions	•Usual care	Quality of Life, including physical and mental health components	None
**10**	Stephens et al. 2021 [53]	Retrospective cohort study	Uganda rural	CDCom program	SBP < 170 mmHg for 6 months. Good adherence. No renal or cardiovascular complications	761 (413 on hypertension treatment)	2016- 2019	•Monthly meetings of patients with VHW and their clinician supervisors at places used for gatherings in the community, delivering integrated care •Content of meetings: treatment monitoring, lifestyle and medication adherence counselling, diagnosis of chronic complications. Referral to health facilities if necessary •BP treatment prioritization according to individual risk and adapted to minimize effects of drug stock outs	•N/A	Diabetes, heart disease, asthma, epilepsy and other NCDs	None
**11**	Otieno et al. 2021 [57]	Prospective cohort study	Kenya and Ghana Urban/rural	-	Hypertensive patients ≥ 18y	1266	2018–2019	•Weekly, bi-weekly, or monthly blood pressure assessments as determined by app or providers at community location, central employment location or home •In-clinic review visits every 30, 60 or 90 days •Digital application-generated personalized educational, supportive, and instructive messages	•N/A	None	-eHealth platform: Empower Health, stores patients’ records -Algorithm driven follow-up provides patients with personalized/risk-based care plans - Platform delivers educational/adherence/locally appropriate healthy lifestyle messages, based on the patient's enrolment risk classification, and follow up
**12**	Vedanthan et al. 2021 [56]	Cluster RCT	Kenya rural	BIGPIC	Hypertensive ≥ 35y patients not on treatment or on treatment < 6 m. No acute illness, non-pregnant or HIV-infected patients	2890	NR	•Monthly meetings in respective groups: -Usual care (UC) plus microfinance (MF) support -Group medical visits (GMV) -Group medical visits plus microfinance support (GMV-MF) •Group medical visits comprised monitoring and counselling	•Usual standard of care	Diabetes	None

The majority were before-after studies, describing post-intervention outcomes [52–54, 57, 58, 60, 62] (7, 58%). Other designs included: case-control [51] (1, 8%), mixed-methods [61] (1, 8%), prospective non-randomized controlled trial [55] (1, 8%), RCT [59] (1, 8%), and cluster RCT [56] (1, 8%). The primary aim of most of the studies was to test a specific intervention adapted to a particular context [51, 55–57, 59–62] (8, 67%), while, four (33%) studies were part of broader health or non-communicable chronic disease (NCD) implementation projects [52–54, 58]. Sample size varied from 42 to 7188 participants, with five (42%) studies including more than 1000 participants [52, 56–58, 62]. The majority of the studies (10, 83%), narrowed the inclusion criteria to participants with uncomplicated aHT [52–56, 58–62]. A total of seven (58%) studies had no comparator arm [52–54, 57, 58, 60, 61], whereas five (42%) provided intra-study comparisons of interventions [51, 55, 56, 59, 62], with either standard of care [51, 55, 59] or a second intervention [56, 62].

### Models of care

Four different service delivery models were described: services provided by community pharmacists [52, 55, 60, 61], temporary or permanent stations placed at strategic and accessible locations in the community [54, 62], routine facility-based care complemented with home visits or services in other community locations to reduce patient visits to the facility [51, 57, 59], and care provided at the time of collecting medication in aHT treatment groups [53, 56, 58]. All models applied different elements of task shifting or task sharing. Medical specialists, including cardiologists or general doctors, had a substantial role in supporting the services in seven (58%) studies, either managing referred patients or supporting the practice of lower cadres [51, 52, 54, 56, 57, 61, 62]. Among non-physician delivered services, aHT care was delivered by nurses [51, 52, 54, 56–59, 62] (8, 67%), community health workers [51–54, 56, 57] (CHW) (6, 50%) or pharmacists [51, 52, 55, 56, 60, 61] (5, 42%). For each model of care, authors described the preparation and training given to the health workers involved. Most commonly, an initial training session included training on BP measurement technique, healthy lifestyle, clinical guidelines, counselling and support techniques, and familiarization with the information capturing tools. Sessions were longer for lay cadres and shorter for health professionals and only three (25%) provided ongoing mentoring or supervision [57, 59, 60].

Five (42%) studies specifically reported on aHT medical treatment regimens [52, 56, 57, 60, 62] (Table 3). Treatment choices reflected historical and context recommendations, as well as, availability of drugs. Most frequently, treatment algorithms used diuretics, calcium channel blockers (CCB), ß-blockers, angiotensin-converting enzyme (ACE) inhibitors, and angiotensin receptor blockers (ARBs). Treatments included the use of monotherapy and combinations, however, none used fixed dose combinations.

**Table 3:** aHT drug regimens used in the included care models

Author and publication year	Reported pharmacological treatment for aHT
**Steyn et al. 1993** [62]	•Men: 25.3% ß-blockers, 22.3% diuretics, 6.4% reserpine-containing preparations •Women: 43.2% diuretics, 15% reserpine-containing preparations, 14.6% ß-blockers
**Oparah et al. 2006** [60]	•Prior to intervention: 33% methyldopa, 11% diuretics, 33% combinations •Intervention: JNC (Joint National Committee) VII guidelines*, 2004
**Ndou et al. 2013** [51]	NR
**Khabala et al. 2015** [58]	NR
**Marfo et al. 2017** [55]	NR
**Nelissen et al. 2018** [61]	NR
**Kuria et al. 2018** [54]	NR
**Adler et al. 2019** [52]	•At enrolment: 36% used a calcium channel blocker (CCB) •After 6-months enrolment: 75.9% patients used diuretics and 69.5% were on a CCB. A total of 24.1% were taking only one medication, 32% were taking two medications and over 30% were taking more than two medications •At 12 m-enrolment: 79.8% were on diuretics, and 71.5% taking a CCB.A total of 23% were taking one medication, 32.6% were taking two medications and over 32% were taking more than two medications
**Bolarinwa et al. 2019 **[59]	NR
**Stephens et al. 2021** [53]	NR
**Otieno et al. 2021** [57]	•A total of 74% of patients were on calcium channel blocker, 64% on ACE or ARB and 14% on diuretics. A minority of patients used other treatments
**Vedanthan et al. 2021** [56]	•Use of diuretics if SBP $\ge$ 140 and < 180 OR DBP $\ge$ 90 and < 110, without edema of legs or dyspnoea on exertion or reduced urine output •Treatment of hypertension in Diabetes: initial ACE inhibitors. Escalate to ARBs with/without diuretics

^*^https://www.nhlbi.nih.gov/files/docs/guidelines/jnc7full.pdf

Seven (58%) studies integrated aHT care with other prevalent chronic health conditions, mostly diabetes [51, 53, 55, 56, 58], and HIV [58]. Only two (16%) models integrated care with other NCDs, such as mental health, epilepsy, asthma, or heart disease [53, 59]. Five (42%) studies used electronic information systems as a substantial component of the model of care, including clinical and computerized decision support systems or e-health platforms [52, 55, 57, 60, 61].

### Measured outcomes

Table 4 summarizes the studies’ outcomes of interest, perceived benefits, and challenges of the models of care, as described by the authors. Two (17%) studies reported on acceptability of the care model [60, 61]; eleven (92%) on BP control [51–53, 55–62]; and 10 (83%) on engagement in care [52–56, 58–62]. Only one (8%) study reported on end-organ damage. Nine (75%) studies report outcomes between an average follow up of 6–12 months [51, 52, 55–61], while 3 (25%) studies reported a follow up longer than one year [53, 54, 57].

**Table 4:** Studies reported outcomes and key findings

	**Author and publication year**	**Country**	**Described outcomes**	**Timeline of outcomes**	**Reported outcomes** (Study comparations if available)	**Key findings reported by authors**	
						**Reported benefits**	**Reported challenges**
**1**	Steyn 1993 [62]	South Africa	•BP control (< 160/95 mmHg) •Engagement in care	4 years	•**BP control:** -In men: SBP decreased by 4,5 mmHg in both intervention towns compared with 1,8 mmHg in the control town -DBP decreased by 1,5 and 2,3 mmHg in control towns, while it increased by 2,2 mmHg in the control town -In women: SBP, mean SBP decreased by 6,3 and 8,0 mmHg in the intervention towns, compared with a decrease of 4,9 mmHg in the control town -DBP decreased by 3,4 and 3,8 mmHg against 0,7 in the control town	•Positive impact on prevention of CVDRFs and BP treatment management	•Limited generalisability due to only inclusion of white population during the Apartheid years •Unclear impact on stand-alone BP intervention, as the program was part of an extensive multifactorial risk factor intervention •Historical BP control targets
**2**	Oparah 2006 [60]	Nigeria	•Acceptability •BP control (< 140/90 mmHg) •Engagement in care	6 months	•**Patient satisfaction:** significantly higher rating than baseline P < 0.0001 •**BP control:** -Significant difference (P < 0.0001) in mean SBP from baseline (187.67 ± 29.46 mmHg) to the end of the study (137.22 ± 21.65 mmHg) -Significant difference (P < 0.0001) in mean DBP from baseline (117.56 ± 21.65) to the end of study (89 ± 17.23) -75% reached SBP goals, while 69% attained DBP goals •**Adherence to treatment**: -Improvement on compliance-rated scores at the end of the study compared to baseline (< 0.006)	•Increased access and acceptability of the BP intervention with the involvement of community pharmacists •Community pharmacists involved in early diagnosis of BP and potential role in CVRFs screening	•Practices in Nigeria do not conform to international standards for community pharmacies •Limited long-term impact due to short follow-up and small sample size •Need to provide remuneration for the community pharmacists
**3**	Ndou 2013 [51]	South Africa	•BP control (< 130/85 mmHg)	8 (2–18) months	•**BP control:** -21.4% of patients in the community were controlled at > 40% of health checks in comparison to 13.1% of clinic patients -In diabetic patients: hypertension was controlled in higher proportion of community-based patients (27.3%) at > 40% of health checks in comparison with 4.8% of clinic patients	•Increased accessibility of services, especially among elder groups •Reduced patient load at the clinics	•Service delivery frequently compromised by lack of doctors, poor drug supply, centralized services, and poor stakeholders coordination •Quality of care compromised by poor management of side effects, lack of CHWs supervision, poor referrals of patients to higher levels, inability to address other determinants of health
**4**	Khabala 2015 [58]	Kenya	•BP control (BP threshold in MACs < 150/100 mmHg) •Engagement in care	12 months	•**BP control:** -A total of 12/2208 consultations were referred back to regular care due to failure to control diabetes/hypertension •**Engagement in care:** -Overall loss to follow-up: 3.5% -LTFU occurred only between the 1st and 2nd MAC attendees -There were no known deaths of MAC patients during the study period •**End-organ damage:** -followed up 211 group participants with creatinine (outcomes not reported)	•Reduced patient burden at clinics •Reduced waiting times and increased appointment flexibility •Free services, leading to increased retention in care	•Unclear impact in long-term outcomes •Very selected population: “Stable”: HIV ≥ 25y on treatment > 6 months (in HIV + > 1y). Criteria of stability: BP < 150/200, HbA1C < 8%, CD4 > 200, undetectable viral load, not WHO stage 3 or 4, or other active disease
**5**	Marfo 2017 [55]	Ghana	•BP control (< 140/90 mmHg/ < 130/80 mmHg in diabetic hypertensive patients) •Engagement in care	6 months	•**BP control:** -Mean SBP difference between the intervention and the control group was statistically significant (p = 0.001) -Mean adherence difference between the two groups was statistically significant (p = 0.001) •**Adherence:** -The intervention group increased in mean adherence scores and the control group showed a decrease in adherence scores at the end of the study. The difference in the mean adherence scores between the two groups was statistically significant	• Increased users satisfaction due to reductions in waiting time and increased access to health education	•Lack of national policies concerning services at community pharmacies •Time consuming intervention for pharmacists (preparing appointments and the preparation of patients reminders) •Remuneration of community pharmacists could increase cost for the patients •Quality of services compromised by lack of assessment of adherence to medicines and poor telecommunication coverage, leading to increased LFU
**6**	Nelissen 2018 [61]	Nigeria	•Acceptability •BP control (SBP < 140/ 90 mmHg in patients < 60y< 150/ 90 mmHg in diabetic and > 60 years) •Engagement in care	6–8 months	•**Acceptability:** -Cardiologists, pharmacists, and patients where content with model of care, however, expressed difficulties with management of mHealth digital platform •**BP control:** -Mean SBP decreased 9.9 mmHg (SD: 18) -BP on target increased from 24 to 56% and an additional 10% had an improved blood pressure. However, this was not associated with duration of mHealth activity •**Engagement in care:** -mHealth activity was present ranging from 38 to 83% across pharmacies - Median mHealth activity duration was 3.3 months. However, patients self-reported more visits than recorded in the mHealth data -52% self-reported low adherence, 24% moderate adherence and 24% high adherence to antihypertensive medication. This distribution did not significantly differ across the pharmacies	•Increased access and quality of care for users •Increased self-care practice and reduction in waiting times •mHealth app bridged the gap between clinicians and pharmacies •Financial savings: costs reductions and ability to negotiate different payment methods with the pharmacists	•Limited representability of population as very selected participants •Patients perspectives: user fees. Sense of being monitored too closely. Unclear links with the cardiologists through the app •Health care workers perspectives: Understaffing. Users fees. Difficulties with connectivity to the mHealth application and usability. Fear of clinicians/cardiologists to have their role been taken over by the pharmacists. Increased workload for clinicians and pharmacies •Overall long-term financial sustainability of the model of care
**7**	Kuria 2018 [54]	Kenya	•Engagement in care	20 months	•**Engagement in care:** -Of the 4960 scheduled follow-up visits, the health facility group were more compliant (64%) than either walkway (60%) or weekend clinic attenders (55%) (P 0.006) -Self-reported adherence of those who complied with scheduled clinic visits was 94%, with walkway at 96%, facility at 94% and weekend at 88%, (P 0.001) -Patients who received hypertension services through the weekend clinic were 76% less likely to adhere to the treatment than those treated at the facility (AOR 0.24, 95% CI 0.10–0.57) -The association between the model of hypertension service delivery and self-reported adherence to medication remained significant even after adjusting for sex and age at enrolment	•Placing full-service clinics in strategic locations to account for travel to work may be effective •Offering services for men outside working hours may increase their participation •Using a simple pill regimen likely increases adherence •Health passports with medical information facilitate long-term care in transient populations	•Services did not provide comprehensive services at a convenient location for patients •Adherence to medication was self-reported and hence could have introduced bias in care •Lack of quality data increased LFU •Compliance with the health facility model was better than in walkway and weekend clinics
**8**	Adler 2019 [52]	Ghana	•BP control (< 140/90 mm Hg) •Engagement in care	6–12 months	•**Blood pressure control:** - 72% (95% CI: 67% to 77%) of participants had their BP under control. SBP was reduced by 12.2 mm Hg (95% CI: 14.4 to 10.1) and diastolic BP by 7.5 mm Hg (95% CI: 9.9 to 6.1) •**Engagement in care:** 552/1339 (41%) patients were in care at 6 m and 338/1339 (25%) were retained in care at 12 months	•Use of Ghana health system existing protocols and medications	•Incomplete picture of medical interventions as ComHIP was connected only with certain HCWs •No control cohort •High LFU rates and staff turnover
**9**	Bolarinwa 2019 [59]	Nigeria	•BP control (< 140/90 mm Hg) •Engagement in care	6–12 months	•**Blood pressure control:** -Mean SBP ± SD (mmHg) was 139.39 ± 23.79 in the intervention group and 140.57 ± 21.90 in the control group (P = 0.658) - Mean DBP ± SD (mmHg) was 86.58 ± 12.11 in the intervention group and 87.27 ± 11.63 in the control group (P = 0.616) •**Engagement in care:** Adherence to treatment was increased in the intervention group (P = < 0.001)	•Improvement of the physical component of quality of life after controlling for the baseline quality of life and age •Possible improvement in adherence linked to improved counselling	•High attrition rates (lower than similar RCTs)
**10**	Stephens 21 [53]	Uganda	•BP control (SBP < 169 mm Hg) •Engagement in care	24 months	•**Blood pressure control:** -Treatment targets: once treatment is initiated for uncomplicated aHT, the target SBP is < 159. If the SBP is 140–169, the patient is given lifestyle advice and followed up regularly by the VHW for a year. If the threshold of SBP > 169 is reached, the patient is enrolled in CDCom -68% hypertensive patients enrolled in CDCom had their most recent blood pressure below the treatment target	•Ability to integrate medical treatment within VHWs screening activities, improving the continuum of care •Services are closer to patients home •VHWs have better rapport with the communities •Increased communication among the different levels of care (primary, secondary and tertiary)	•Inconsistency in measuring BP, leading to over/under measurement •Increased cost if there is not a comprehensive package of care •Rotation of clinical staff and lack of clear job descriptions •Drug stock-outs •Cost of drugs (user fees) likely rends the model unsuccessful
**11**	Otieno 21 [57]	Kenya and Ghana	•BP control (< 140/90 mm Hg)	7–16 months	•**Blood pressure control:** -SBP decreased significantly through 12 months in both the overall cohort (− 9.4 mmHg, p < .001) and in the uncontrolled subgroup (− 17.6 mmHg, p < .001) -Proportion of patients with controlled pressure increased from 46% at baseline to 77% at 12 months (p < .001)	•Co-created, locally appropriate model of care implemented to address formidable socioeconomic barriers •The drops BP plateaued at about 4 months and were sustained over the 12-month follow-up period •In-clinic patient visits were reduced 60% as compared to standard monthly visits	•Limited representability, as cohort may not represent correspond to the broader sub-population of undiagnosed or untreated patients outside an organized health care system •The analysis did not include a control arm for comparison. However, the magnitude of the BP reduction and the sustainment of the large reduction through a year of follow-up provided evidence against the effect of a Hawthorne effect
**12**	Vedanthan 2021 [56]	Kenya	•BP control (< 140/90 mm Hg) •Engagement in care	12 months	•**Blood pressure control:** -Model-based estimates showed that, compared with the UC arm, the mean reduction in SBP was 3.9 mm Hg greater in the GMV-MF arm (98.3% CI: -8.5 to 0.7 mm Hg; p = 0.05), 3.3 mm Hg greater in the GMV arm (98.3% CI: -7.8 to 1.2 mm Hg; p = 0.09), and 2.3 mm Hg greater in the MF arm (98.3% CI: -7.0 to 2.4 mm Hg; p = 0.25) •**Engagement in care:** - 12-moths retention: 661/708 (93%) UC, 673/709 (95%) MF, 704/740 (95%) GMVs, 672/763 GMV-MF (88%)	•Observed improvements in BP control were clinically meaningful and would yield substantial long-term cardiovascular and mortality benefit •Model of care addressing social determinants of health	•Contingent upon enrolment led to differential exposure to the intervention across participants •Follow-up duration was insufficient to demonstrate a significant benefit •Unlikely, but possible cross-contamination across the trial arms •Study population not fully representative of the general population. However, the economic challenges experienced by study participants were not dissimilar from a large proportion of the global population

Acceptability was reported either collecting the experience of the health workers [61], or measuring patients’ satisfaction through qualitative research and the Larson satisfaction questionnaire [63]. Of the two studies reporting satisfaction, one model delivered home-based aHT treatment and one provided care in community pharmacies [60, 61]. Participants reported benefits in adhering to the treatment and general knowledge on self-care practices.

With regards to reporting BP control, targets for (SBP, (DBP and aHT definitions varied, reflecting historical definitions [62] or pragmatic targets linked to inclusion criteria in the care model [53, 58]. Seven (58%) studies [52, 55–57, 59–61] used SBP ≤ 140 mmHg and DBP ≤ 90 mmHg to define BP control, while two (16%) [55, 61] modified control thresholds for diabetic patients. Eight (67%) studies showed a significant improvement in BP control [51, 52, 55, 57, 58, 60–62] and one (8%) showed that BP was controlled in higher proportion for diabetic patients receiving community-based services [51].

Eight (67%) studies reported engagement in care [51, 52, 54–56, 58, 59, 61] using different measures: lost to follow-up or death [54, 58], self-reported adherence to the treatment [54, 55, 59–61], regular use of the e-health support platform [61] or attendance to follow up visits [51, 52, 56, 58]. Two (16%) community pharmacy models in West Africa were the only ones that reported significant improvements in engagement in care and adherence to aHT treatment [55, 59], whereas two studies suggest that community care posts and home-based care could increase long-term engagement in aHT care [52, 54].

The only study reporting end-organ damage measured serum creatinine as surrogate marker for renal function. In this care model, laboratory tests were offered at the time of patients group meetings and collection of aHT medication in a subset of participants [58].

Authors reported the perceived benefits of the aHT models of care in relation to the health system and the users. Benefits for the health system included: task-sharing across different professionals, decrease in daily patients load at facilities, and possibility to offer wider access for services and prevention of other CVDs [51, 53–62]. Perceived advantages from the patients’ perspective referred to increased flexibility to access services, and reduction of costs and waiting times [51, 53–55, 57, 58, 61]. One study noted positive impacts in patients’ quality of life, as the model of care addressed broader social determinants of health closely linked to CVDs, such as poverty, rather than just providing aHT treatment [56].

Authors also reported the weaknesses of these aHT care models. Doubts on generalizability of the models arose in relation to strict inclusion criteria, as care was provided either to selected groups or clinically stable participants. Seven (7, 58%) used clinically narrow eligibility criteria, excluding patients with complicated aHT, severe conditions, or comorbidities [53, 54, 56–58, 61]. One study in South Africa provided care only to the privileged white population during Apartheid [62]. High attrition rates through lost to follow-up and mortality, deficiencies in data quality, small sample sizes, short follow-up periods, or lack of control arms compromised the report of accurate outcomes and the capacity to provide a more complete picture of the real benefit of these models [52–55, 57, 59, 60, 62]. Poor sustainability of care models was brought up in relation to the use of vertical, non-integrated interventions, including parallel remuneration of health workers, lack of staff, medication stock outs or difficulties in managing and sustaining eHealth solutions [51–53, 55, 60, 61]. The overall quality of provided services was a common concern to authors, including difficulties in providing ongoing supervision and mentoring of lower cadres [51–55]. Specifically, the models testing services at community pharmacies in West Africa expressed concerns about the capacity to contribute to a substantial change in service delivery, as the strategy was too far away from existing policies and standards, and sustainability was heavily associated with motivation and remuneration of professionals [55, 60].

### Quality of evidence

Nine cohort studies and one case control study were evaluated using the Newcastle–Ottawa scale [64–66] (Table 5). All studies scored below 6, mainly driven by very narrowly selected study populations and the absence of comparators in many of the studies. One RCT and one cluster-RCT were assessed using the Cochrane Collaboration’s tool for assessing risk of bias in RCTs [48, 49] (Table 6). Overall bias assessment was “low risk for bias” for one study and “some concerns” for the other.

**Table 5:** Risk of bias assessment for cohort and case–control studies (Newcastle–Ottawa Scale)

Author and publication year	Selection				Comparability	Outcome			Score	Quality
	1	2	3	4		1	2	3		
Steyn et al. 1993 [62]	**-**	**-**	**✷**	**✷**	**✷**	**✷**	**✷**	**-**	5	high risk of bias
Oparah et al. 2006 [60]	**-**	**-**	**✷**	**✷**	**-**	**✷**	**✷**	**85%✷**	5	high risk of bias
Ndou et al. 2013 [51]	**-**	**✷**	**✷**	**✷**	**✷**	**✷**	**-**	**-**	5	high risk of bias
Khabala et al. 2015 [58]	**-**	**-**	**✷**	**-**	**-**	**✷**	**-**	**-**	2	high risk of bias
Marfo et al. 2017 [55]	**-**	**✷**	**✷**	**✷**	**-**	**✷**	**✷**	**-**	5	high risk of bias
Nelissen et al. 2018 [61]	**-**	**-**	**✷**	**✷**	**-**	**✷**	**✷**	**-**	4	high risk of bias
Kuria et al. 2018 [54]	**-**	**-**	**✷**	**✷**	**-**	**-**	**✷**	**-**	3	high risk of bias
Adler et al. 2019 [52]	**-**	**-**	**✷**	**✷**	**-**	**✷**	**✷**	**-**	4	high risk of bias
Stephens et al. 2021 [53]	**✷**	**-**	**✷**	**✷**	**-**	**✷**	**✷**	**-**	5	high risk of bias
Otieno et al. 2021 [57]	**-**	**-**	**✷**	**-**	**-**	**✷**	**✷**	**✷**	4	high risk of bias

**Table 6:** Risk of bias assessment for single arm and cluster randomized trials (Cochrane Collaboration’s tool)

Author and publication year	Bias arising from the randomization process	Bias arising from the timing of identification and recruitment of individual participants in relation to timing of randomization	Bias due to deviations from intended interventions	Bias due to missing outcome data	Bias in measurement of the outcome	Bias in selection of the reported result	Overall bias
Bolarinwa et al. 2019 [59]	Low risk	-	Low risk	Low risk	Low risk	Low	Low risk
Vedanthan et al. 2021 [56]	Low risk	Some concerns	High risk	Low risk	Low risk	Low risk	Some concerns

^*^The assessment was conducted using the Revised Cochrane risk-of-bias tool for randomized trials and cluster randomized trials (RoB 2) (https://sites.google.com/site/riskofbiastool/welcome/rob-2-0-tool?authuser=0). Scoring was assigned following the algorithms in guidance documents

## Discussion

To our knowledge, this scoping review is the first comprehensive analysis of community-based aHT treatment models in sub-Saharan Africa. We systematically compiled and synthesized the current evidence related to service delivery models for aHT treatment that differ from traditional, facility-based care between 1993 and 2021. The increasing number of publications in recent years indicates that this is an active field where rapid developments can be expected in future [67–71]. We identified 12 studies that described one or more outcomes of interest from four distinct types of community-based aHT service delivery in five countries. However, only a minority of studies (4, 33%) compared alternative models to conventional care or to other interventions, making it difficult to draw solid conclusions about the overall effectiveness of these models on clinical outcomes. Due to the wide heterogeneity of the models of care, inclusion criteria, outcome definitions, participants follow up and study types we only described each of the studies individually, rather than providing aggregated statistics.

**Fig. 4:**
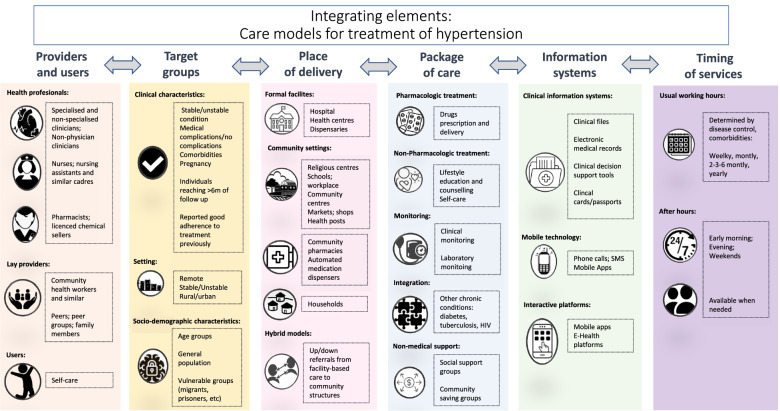
A framework for design of aHT service models, considering their building blocks or main integrating elements

In the process of summarizing the literature for this scoping review, we abstracted the main elements that integrate the models of care, as described by the authors (manuscript tables). These elements constitute the “building blocks” of each care model and are represented in Fig. 4: cadre of health care provider (who delivers the service, including self-care), target population (for whom the care model is created), location of service delivery (where is the service provided), components of the service package, information systems (methods used for collecting information about the users and the service), and the timing of service delivery (when is the service available to the user). Each of these elements is intrinsically composed by other components. i.e.: in the “information systems” category, different models use either paper-based/digitalized patients’ files/cards and/or digital technology. We propose the use of these building blocks to either design or analyze care models for aHT and in general CVDs. To tailor a model of care to a given setting, each of the six blocks should be taken into account and adapted, considering different aspects, such as: setting, resources, cultural preferences, or specific needs of the target population (Fig. 4). Similar models have been used to scale up tuberculosis or HIV services [29, 72–76].

The West African experiences mostly integrated pharmacists and microfinance solutions in urban areas, while East and Southern African models tested interventions that increased access to care in rural communities. In all service models, care is most often provided by lower cadres of health workers, decreasing frequency of interactions with routine services, and combining a high level of self-care. However, most of the models only included participants with already an acceptable BP control, had a short follow-up period, and failed to provide comparable performance with facility-based care in the same setting. Although it is hard to evaluate their real impact, the reported care models do not seem to be associated with lower user’s satisfaction or worse treatment outcomes.

Beyond clinical indicators that report individual aHT treatment outcomes for participants receiving care in these models, a few studies collected patients’ and service providers’ perspectives. Future studies should seek a combination of quantitative and qualitative data and possibly socio-economic data to understand the real reach and impact of such models. The use of electronic information systems becomes an important part of the care in the most recent studies. Patients receive reminders for adherence to medication, general lifestyle counseling or provision of personalized risk-based care plans through, phone calls, SMS, or use of other e-Health platforms. Similarly, these systems support communication between medical specialists, nurses and lay workers [52, 53, 55, 56, 60, 61].

One interesting finding of this review reinforces the idea that expanding the provision of chronic aHT care, and probably other chronic health conditions, to health workers and structures that are outside of traditional care in facilities, reduces, but does not eliminate the need of care provided by medical doctors or specialized nurses. Rather, these services can be used to provide referral paths to manage patients that need to be evaluated for more complex comorbidities or new cardiovascular events by specialised health workers. Our findings describe diverse and heterogenous models of care and suggest that each setting requires its own specifically adapted model of care, taking into account the six building blocks after careful analysis of local gaps and needs. As such, there is not just “one size fits all” care model to efficiently expand out-of-clinic aHT management.

In an effort to close the global aHT treatment gap and improve BP control at societal level, in 2021 the WHO issued updated guidelines for the pharmacological treatment of aHT in non-pregnant adults [3]. These new global recommendations guide decisions such as the threshold for the initiation of pharmacological aHT treatment, choice of treatment regimens and drug combinations, frequency of patients follow up, BP control targets, and the cadre of providers who may initiate or manage long-term treatment. However, regarding frequency of patients’ follow up and treatment by nonphysician professionals these recommendations remain conditional due to low-certainty evidence. Through scoping the published literature on this topic, we have identified additional research questions where future research could help establishing evidence-based recommendations to scale up similar aHT models of care. First, the development of standard descriptions of the models of care, taking into account the six building blocks, definitions of inclusion criteria of participants and clinical outcomes will be needed. Second, as aHT is a chronic condition, it will be important to understand the use of these models to achieve and maintain BP control beyond the first 12 or 24 months of enrolment, and even longer follow up. Third, to understand the potential of these models to improve BP control at population level, it will be important to describe the patterns of transition between conventional aHT care and one or subsequent alternative models across years of care. Fourth, investigators could provide a description of the capacity that each model of care has to reach BP targets after a period of uncontrolled BP and to integrate care for important co-morbidities, like diabetes, HIV, or tuberculosis. Fifth, reports should aim to demonstrate a decrease of overall risk in CVD events and aHT-related end-organ damage. Sixth, studies should also include a description of the wider hypertensive population, not included in these models, including their treatment outcomes for reference comparison. Lastly, the reporting of costs and cost-effectiveness will be crucial to mobilize investments that can catalyse a significant scale up of these services.

### Strengths and limitations

Our review provides a comprehensive description and evaluation of the published community-based aHT care models, following a structured methodological framework. Equally, this review has several limitations. The concept of non-traditional, outside-of-facility health service is heterogenous, poorly defined and lacks standard terminology. Our search terms included most common related synonyms, however, despite the efforts to develop a broad literature search strategy following PRISMA guidance, the selection of standard search terms and databases may have excluded some relevant publications. Our search also excluded regional databases and grey literature; therefore, it is possible that we have missed evidence provided by interventions used in practice and not published. Lastly, we could have missed relevant data when the authors failed to provide sufficient details or disaggregated results [69, 77–80].

## Conclusions

The search for efficient and sustainable service delivery models for the management of aHT in sub-Saharan settings, outside of conventional care, is a rapid evolving field. This scoping review has identified different community-based models that can potentially be seeds of scalable programs that integrate comprehensive chronic care.

However, the wide heterogeneity of the studies, lack of standardization of definitions and measurement of outcomes, small number of participants, short follow up, and lack of reliable comparisons with standard of care, does not allow to describe their real impact in achieving long-term BP control and overall CVD risk decrease. The available literature does not provide a sound basis for policymakers and implementers on whether, and in what form, community-based care delivery models for aHT could be applied to counteract the growing CVD burden in sub-Saharan Africa. We propose that future projects and studies implementing and assessing community-based models of aHT care are designed and described according to six building blocks defining the providers, target groups, components, location, time of services and their use of information systems.

## Supplementary Information

**Additional file 1: Annex 1.** ScR databases search strategy.

## Data Availability

All data generated or analysed during this study are included in this published article [and its supplementary information files]. All articles used for the review are in the references section.

## Abbreviations

ACE: Angiotensin-Converting Enzyme
aHT: Arterial Hypertension
ARBs: Angiotensin Receptor Blockers
BP: Blood Pressure
CCB: Calcium channel blockers
CINAHL: Cumulative Index to Nursing and Allied Health Literature
CVDs: Cardiovascular Diseases
CHW: Community Health Worker
DBP: Diastolic Blood Pressure
NCDs: Non-Communicable Diseases
RCTs: Randomized Controlled Trials
SBP: Systolic Blood Pressure
