# Correction: Community-based care models for arterial hypertension management in non-pregnant adults in sub-Saharan Africa: a literature scoping review and framework for designing chronic services

**DOI:** 10.1186/s12889-022-13988-y

**Published:** 2022-09-05

**Authors:** Lucia González Fernández, Emmanuel Firima, Elena Robinson, Fabiola Ursprung, Jacqueline Huber, Alain Amstutz, Ravi Gupta, Felix Gerber, Joalane Mokhohlane, Thabo Lejone, Irene Ayakaka, Hongyi Xu, Niklaus Daniel Labhardt

**Affiliations:** 1 Department of Medicine, Swiss Tropical and Public Health Institute, 4051, Allschwil, Basel, Switzerland; 2 University of Basel, 4001 Basel, Switzerland; 3 SolidarMed, Partnerships for Health, Maseru, 0254 Lesotho; 4 Faculty of Biomedical Sciences, Università Della Svizzera Italiana, 6900 Lugano, Switzerland; 5 Swiss TPH Library, Swiss Tropical and Public Health Institute, 4051, Allschwil, Basel, Switzerland; 6 Non-Communicable Diseases Unit, Ministry of Health, Maseru, 174 Lesotho; 7 Department of Non-Communicable Diseases, World Health Organization, 1202 Geneva, Switzerland; 8 Department of Infectious Diseases and Hospital Epidemiology, University Hospital Basel, 4031 Basel, Switzerland

**Correction: BMC Public Health 22, 1126 (2022)**

**https://doi.org/10.1186/s12889-022-13467-4**

The original publication of this article [1] contained an erroneous label in figure 3 titled “Bubble size”. The original article has been updated to correct the label.
