# Supplementary material for: Community-based care models for arterial hypertension management in non-pregnant adults in sub-Saharan Africa: a literature scoping review and framework for designing chronic services
Source: BMC Public Health. 2022 Jun 4;22:1126. doi: 10.1186/s12889-022-13467-4 (PMC9167524; doi:10.1186/s12889-022-13467-4)
Supplement: Supplementary file 1 — Additional file 1: Annex 1. ScR databases search strategy. [file 12889_2022_13467_MOESM1_ESM.docx]

*Annex 1: ScR databases search strategy*

**Search strategy**

The search strategy was developed to simultaneously conduct two scoping reviews looking at community-based models to treat hypertension (aHT) and diabetes mellitus (DM). Due to this, we combined the terms looking at the population concept. We developed terms that defined concepts according to the PICO structure (PMID: 7582737): Population (condition and geographic location) and Intervention (community-based models of care).


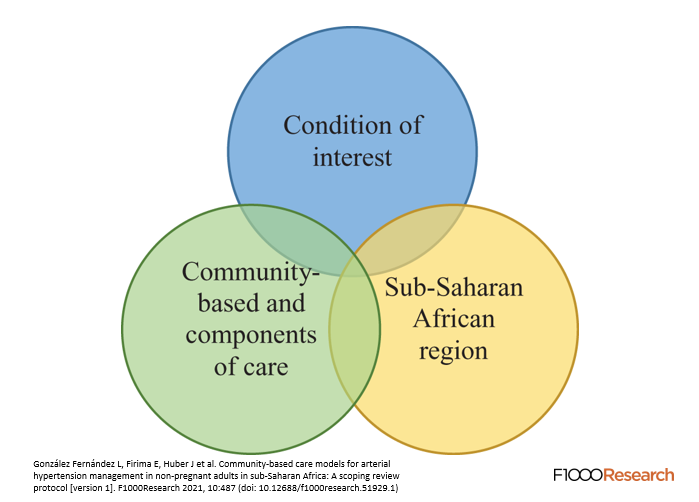


The foundation of the disease part of the population concept was laid in a literature searching workshop and further developed (<https://f1000research.com/articles/10-487/v1>). We developed the geographic part with a combination of already available search filters as well as the help of several websites (Worldbank, nationsonline, CIA). The string related to the geographical component was developed based on Campbell et al (<https://docs.google.com/document/d/1OD6i8JGEexxtjKRhYLs59MrjGPL_W-eE2b6Zzej0BKU/edit?usp=embed_facebook>) and the United Nations standard country or area codes for statistical use (<https://unstats.un.org/unsd/methodology/m49/> ).

For the community concept an initial limited search of Embase was undertaken to identify articles on the topic. The text words contained in the titles and abstracts of relevant articles, and the index terms used to describe the articles were used to develop the search strategy. The string related to the components of the care models (“who”, “where”, “how”) but terms such as “health programme”, “adherence”, “medical assistance” where excluded from the string design, as, they increased its complexity and the translation to other databases. In addition, broad terms for treatment and care were combined with specific community-based terms. The full search strategy was first drafted for the databases Embase Elsevier and subsequently translated to the other databases. Studies published in any language or at any date are included but limitations to conference abstracts and juveniles were applied for Embase and MEDLINE Ovid. Macros developed by Erasmus MC information specialists (PMID: 30271302) were used to translate the search string to Medline Ovid. The tool SR-Accelerator (<https://research.bond.edu.au/en/publications/systematic-review-amp-meta-analysis-automation-tools-to-help-your>) was used to translate the Medline Ovid search string to Scopus and CINAHL databases. As these tools only apply the correct for the corresponding database, the thesaurus terms had to be adapted manually. Scopus was only searched via text words, as indexed keywords (index terms) are chosen by content suppliers and not uniformly applied.

**Embase Search string**

('diabetes mellitus'/exp OR 'insulin resistant diabetes mellitus'/exp OR 'diabetic patient'/exp OR 'diabetic complication'/exp OR 'diabetes education'/exp OR 'diabetes educator'/exp OR 'blood glucose monitoring'/exp OR 'glycemic control'/exp OR 'glycemic index'/exp OR 'glycemic load'/exp OR 'hyperglycemia'/exp OR 'dysglycemia'/exp OR diabet*:ab,ti OR antidiabet*:ab,ti OR 'dm 2':ab,ti OR 'dm2':ab,ti OR 'dm type 2':ab,ti OR 'dm type2':ab,ti OR niddm:ab,ti OR t2dm:ab,ti OR 't2 dm':ab,ti OR ((glycemic OR glycaemic OR glycemia OR glycaemia OR glycemie) NEXT/2 (control OR index OR value OR load)):ab,ti OR hyperglycemi*:ab,ti OR hyperglycaemi*:ab,ti OR hyperglucemi*:ab,ti OR hyper-glycemi*:ab,ti OR hyper-glycaemi*:ab,ti OR hyper-glucemi*:ab,ti OR dysglycemi*:ab,ti OR ((high OR elevated OR monitor*) NEAR/3 ('blood glucose' OR 'blood sugar' OR 'plasma glucose' OR 'plasma sugar' OR 'serum glucose' OR 'serum sugar')):ab,ti OR 'elevated blood pressure'/exp OR 'blood pressure'/de OR 'arterial pressure'/exp OR 'systolic blood pressure'/de OR 'abnormal blood pressure'/de OR 'blood pressure measurement'/exp OR 'blood pressure monitor'/exp OR 'blood pressure monitoring'/exp OR 'hypertension complications'/exp OR 'hypertensive patient'/exp OR 'antihypertensive therapy'/exp OR (hypertens* OR 'hyper tens*' OR 'blood pressur*' OR 'arterial pressur*' OR 'vascular pressur*' OR 'intravascular pressur*' OR 'blood tension' OR 'arterial tension' OR 'vascular tension' OR 'intravascular tension' OR antihypertensive):ab,ti OR 'insulin resistance'/exp OR 'insulin resistance index'/exp OR 'metabolic syndrome*':ab,ti OR 'insulin resistance':ab,ti)

AND

('africa south of the sahara'/exp OR 'africa south of the sahara':ab,ti OR 'sub-sahara* africa*':ab,ti OR 'subsahara* africa*':ab,ti OR 'black africa*':ab,ti OR 'west africa'/exp OR 'west african'/exp OR 'west* africa*':ab,ti OR 'east african'/exp OR 'east* africa*':ab,ti OR 'southern african'/exp OR 'south* africa*':ab,ti OR 'central african'/exp OR 'central africa*':ab,ti OR 'equatorial africa*':ab,ti OR 'middle africa'/exp OR 'middle africa*':ab,ti OR angola*:ab,ti OR benin*:ab,ti OR dahomey:ab,ti OR botswana*:ab,ti OR bechuanaland:ab,ti OR 'burkina faso':ab,ti OR burkin*:ab,ti OR 'upper volta':ab,ti OR burundi*:ab,ti OR urundi:ab,ti OR cameroon*:ab,ti OR camerun:ab,ti OR kamerun:ab,ti OR cameroun:ab,ti OR 'cape verde*':ab,ti OR 'cabo verde':ab,ti OR centrafri*:ab,ti OR 'ubangi-shari':ab,ti OR 'oubangi-shari':ab,ti OR chad*:ab,ti OR tchad:ab,ti OR comoro*:ab,ti OR comores:ab,ti OR comoran:ab,ti OR comorian:ab,ti OR congo*:ab,ti OR kongo:ab,ti OR zaire:ab,ti OR 'cote d`ivoire':ab,ti OR 'ivory coast':ab,ti OR ivorian*:ab,ti OR djibouti*:ab,ti OR 'afars and issas':ab,ti OR eritrea*:ab,ti OR eswatini:ab,ti OR swazi*:ab,ti OR ethiopia*:ab,ti OR abyssinia:ab,ti OR gabon*:ab,ti OR gabun:ab,ti OR gambia*:ab,ti OR senegambia:ab,ti OR ghana*:ab,ti OR 'gold coast':ab,ti OR guinea*:ab,ti OR guinée:ab,ti OR guiné:ab,ti OR 'bissau-guinean':ab,ti OR equatoguinean:ab,ti OR kenya*:ab,ti OR lesotho*:ab,ti OR basutoland:ab,ti OR liberia*:ab,ti OR madagasca*:ab,ti OR malagasy*:ab,ti OR malawi*:ab,ti OR nyasaland:ab,ti OR mali:ab,ti OR malian*:ab,ti OR mauritania*:ab,ti OR mauritanie:ab,ti OR mauritius:ab,ti OR 'république de maurice':ab,ti OR mayotte:ab,ti OR mahoran*:ab,ti OR mozambi*:ab,ti OR mocambique:ab,ti OR namibia*:ab,ti OR niger*:ab,ti OR réunion:ab,ti OR réunionese:ab,ti OR rwanda*:ab,ti OR ruanda*:ab,ti OR senegal*:ab,ti OR 'seychellene'/exp OR seychell*:ab,ti OR 'sierra leon*':ab,ti OR somali*:ab,ti OR 'sudanese'/exp OR sudan*:ab,ti OR tanzania*:ab,ti OR tansania*:ab,ti OR tanganyika:ab,ti OR zanzibar:ab,ti OR togo*:ab,ti OR uganda*:ab,ti OR zambia*:ab,ti OR sambia*:ab,ti OR zimbabwe*:ab,ti OR rhodesia:ab,ti OR luanda:ab,ti OR lubango:ab,ti OR cabinda:ab,ti OR 'porto-novo':ab,ti OR cotonou:ab,ti OR 'abomey-calavi':ab,ti OR gaborone:ab,ti OR ouagadougou:ab,ti OR 'bobo-dioulasso':ab,ti OR bujumbura:ab,ti OR yaounde:ab,ti OR douala:ab,ti OR praia:ab,ti OR bangui:ab,ti OR n`djamena:ab,ti OR ndjamena:ab,ti OR moroni:ab,ti OR brazzaville:ab,ti OR 'pointe-noire':ab,ti OR kinshasa:ab,ti OR 'mbuji-mayi':ab,ti OR lubumbashi:ab,ti OR kananga:ab,ti OR kisangani:ab,ti OR bukavu:ab,ti OR yamoussoukro:ab,ti OR abidjan:ab,ti OR malabo:ab,ti OR asmara:ab,ti OR mbabane:ab,ti OR 'addis ababa':ab,ti OR libreville:ab,ti OR banjul:ab,ti OR accra:ab,ti OR kumasi:ab,ti OR 'sekondi takoradi':ab,ti OR conakry:ab,ti OR bissau:ab,ti OR nairobi:ab,ti OR mombasa:ab,ti OR mombassa:ab,ti OR maseru:ab,ti OR monrovia:ab,ti OR antananarivo:ab,ti OR lilongwe:ab,ti OR 'blantyre-limbe':ab,ti OR bamako:ab,ti OR nouakchott:ab,ti OR 'port louis':ab,ti OR maputo:ab,ti OR matola:ab,ti OR nampula:ab,ti OR windhoek:ab,ti OR niamey:ab,ti OR abuja:ab,ti OR lagos:ab,ti OR kano:ab,ti OR ibadan:ab,ti OR 'port harcourt':ab,ti OR kigali:ab,ti OR 'sao tome*':ab,ti OR dakar:ab,ti OR freetown:ab,ti OR mogadishu:ab,ti OR hargeisa:ab,ti OR hargeysa:ab,ti OR pretoria:ab,ti OR 'cape town':ab,ti OR johannesburg:ab,ti OR Soweto:ab,ti OR durban:ab,ti OR 'port elizabeth':ab,ti OR 'west rand':ab,ti OR juba:ab,ti OR khartoum:ab,ti OR nyala:ab,ti OR 'dar es salaam':ab,ti OR dodoma:ab,ti OR mwanza:ab,ti OR lome:ab,ti OR kampala:ab,ti OR lusaka:ab,ti OR harare:ab,ti)

AND

('decentralization'/exp OR 'telehealth '/exp OR 'telemetry'/exp OR 'teleophthalmology'/exp OR Telehealth:ab,ti OR Tele-health:ab,ti OR Telemedic*:ab,ti OR Tele-medic*:ab,ti OR telemetry:ab,ti OR tele-metry:ab,ti OR biotelemetry:ab,ti OR radiotelemetry:ab,ti OR teleradiometry:ab,ti OR telesensing:ab,ti OR tele-sensing:ab,ti OR telemonitor*:ab,ti OR tele-monitor*:ab,ti OR telenurs*:ab,ti OR tele-nurs*:ab,ti OR teleconsultat*:ab,ti OR tele-consultat*:ab,ti OR telerehabilitation*:ab,ti OR tele-rehabilitation*:ab,ti OR tele-ophthalmology:ab,ti OR teleophthalmology:ab,ti OR ((virtual OR remote OR distance OR distant OR e) NEXT/2 (medic* OR monitor* OR nurs* OR consultat* OR rehabilitation* OR support* OR counsel* OR treatment* OR therap* OR sensing)):ab,ti OR (emedic* OR emonitor* OR enurs* OR econsultat* OR erehabilitation OR esupport* OR ecounsel* OR etreatment* OR etherap* OR esensing):ab,ti OR Mhealth:ab,ti OR M-health:ab,ti OR 'mobile health':ab,ti OR Ehealth:ab,ti OR E-health:ab,ti OR 'e-counseling'/exp OR 'e-mail'/exp OR 'hotline'/exp OR 'interactive voice response system'/exp OR 'internet'/de OR 'web-based intervention'/exp OR 'mobile phone'/exp OR 'telephone'/exp OR 'social media'/exp OR 'telecommunication'/exp OR 'text messaging'/exp OR 'videoconferencing'/exp OR 'web conferencing'/exp OR 'webcast'/exp OR 'podcast'/exp OR 'podcasting'/exp OR 'webinar'/exp OR 'mobile application'/exp OR 'website'/exp OR 'mobile technology'/exp OR 'e mail*':ab,ti OR 'email*':ab,ti OR (electronic NEXT/2 (mail* OR messaging)):ab,ti OR hotline*:ab,ti OR 'interactive voice response system*':ab,ti OR ((web OR online OR on-line OR internet OR video OR virtual OR tele) NEAR/3 (intervention* OR conferenc* OR communication OR seminar* OR information*)):ab,ti OR internet:ab,ti OR 'world wide web':ab,ti OR www:ab,ti OR ((mobile OR cell OR smart OR portable) NEXT/2 (phone* OR telephone*)):ab,ti OR 'social media*':ab,ti OR facebook:ab,ti OR twitter:ab,ti OR telecommunication*:ab,ti OR broadcasting:ab,ti OR 'text messag*':ab,ti OR texting:ab,ti OR teleconferenc*:ab,ti OR videoconferenc*:ab,ti OR webconferenc*:ab,ti OR webcast*:ab,ti OR podcast*:ab,ti OR webinar*:ab,ti OR SMS:ab,ti OR telephone*:ab,ti OR smartphone*:ab,ti OR ((mobile OR portable OR tablet) NEXT/2 (app OR apps OR application*)):ab,ti OR website:ab,ti OR homepage:ab,ti OR 'mobile technology':ab,ti OR 'ambulatory care'/exp OR decentrali*:ab,ti OR ((ambulatory OR dispensary OR extramural OR extra-mural OR outpatient OR 'outpatient health*' OR out-patient OR 'out-patient health*' OR out-of-office) NEXT/2 (care OR healthcare OR service* OR setting* OR monitoring* OR treatment* OR therap*)):ab,ti OR 'community care'/de OR 'community based rehabilitation'/exp OR 'community health nursing'/exp OR 'community integration'/exp OR 'community program'/exp OR 'community medicine'/exp OR 'community support'/exp OR 'community based distribution'/exp OR 'task shifting'/exp OR 'task sharing'/exp OR ((community OR 'community health*' OR district OR 'public health' OR nurse-led OR pharmacist-led OR nurse-based OR pharmacist-based OR 'social service' OR collaborative) NEXT/3 (care OR healthcare OR nurs* OR integration OR service* OR program* OR medicine OR support* OR engagement OR intervention* OR deliver* OR outreach OR distribution OR network* OR 'participatory method*' OR rehabilitation* OR resource* OR strateg* OR project* OR approach* OR testing OR screening*)):ab,ti OR 'task shar*':ab,ti OR 'task shift*':ab,ti OR 'home care'/exp OR 'home monitoring'/exp OR 'residential care'/exp OR 'adult day care'/exp OR ((adult OR home OR domicil* OR domestic OR visiting OR residential OR residence) NEAR/3 (care OR healthcare OR nurs* OR help OR assistance OR service* OR treatment* OR therap* OR 'medication review*' OR pharmac* OR monitor* OR visit* OR agenc* OR testing OR screening* OR diagnos*)):ab,ti OR homecare:ab,ti OR 'house call*':ab,ti OR 'home diagnostic test'/exp OR 'housebound patient*':ab,ti OR 'adult day care':ab,ti OR 'family service'/exp OR 'family service*':ab,ti OR 'family involve*':ab,ti OR 'family based':ab,ti OR 'family-based' :ab,ti OR 'family health*':ab,ti OR 'outpatient care'/exp OR 'out-of-facilit*':ab,ti OR 'health center'/exp OR ((health OR 'health service' OR sanitary) NEXT/2 (center* OR centre* OR clinic* OR institute* OR unit* OR resort* OR facility OR facilities)):ab,ti OR 'pharmacy (shop)'/de OR 'mail order pharmacy'/exp OR 'online pharmacy'/exp OR 'apothecary':ab,ti OR 'chemist shop*':ab,ti OR 'chemist`s shop*':ab,ti OR 'pharmacies':ab,ti OR 'pharmacy':ab,ti OR 'pharmaceutical service*':ab,ti OR 'rural health care'/exp OR 'school health service'/de OR 'school health nursing'/exp OR ((rural OR school* OR workplace OR worksite OR work-place OR work-site) NEAR/3 (health* OR care OR medicine OR nurse OR nurses OR nursing OR intervention* OR program* OR approach* OR service* OR strateg*)):ab,ti OR 'self care'/exp OR 'self monitoring'/exp OR (self NEXT/2 (care* OR help OR management OR monitoring OR treatment* OR medication OR nurturance OR testing OR measur*)):ab,ti OR 'selfcare':ab,ti OR 'selfhelp':ab,ti OR 'selfmanagement':ab,ti OR 'selfmonitor*':ab,ti OR 'selftreatment':ab,ti OR 'selfmedication*':ab,ti OR 'social network'/exp OR 'social care'/exp OR ((social OR spiritual) NEXT/2 (care OR support OR network* OR service* OR work)):ab,ti OR 'support group'/exp OR 'support group*':ab,ti OR 'support program*':ab,ti OR paramedical:ab,ti OR para-medical:ab,ti OR non-health:ab,ti OR non-healthcare:ab,ti OR non-medical:ab,ti OR ((health OR healthcare) NEXT/2 ('support worker*' OR assistant* OR aid*)):ab,ti OR ((community OR district OR 'public health' OR assistan* OR aid* OR staff* OR manpower OR personnel) NEAR/4 (nurse OR nurses OR nursing OR matron*)):ab,ti OR 'midlevel health professional*':ab,ti OR 'midlevel health provider*':ab,ti OR 'paramedical personnel'/de OR 'pharmacy technician'/exp OR 'health practitioner'/exp OR 'health visitor'/exp OR 'nurse'/exp OR 'nursing assistant'/exp OR 'nursing staff'/exp OR 'paramedical profession'/exp OR 'pharmacist'/de OR 'community pharmacist'/exp OR 'health auxiliary'/exp OR ((community OR 'community based' OR village OR lay OR 'allied health' OR 'auxiliary health' OR 'health auxiliary') NEXT/3 (worker* OR provider* OR personnel OR profession* OR staff OR aide* OR supporter* OR volunteer* OR advocate* OR team* OR group* OR pharmacist*)):ab,ti OR 'barefoot doctor*':ab,ti OR 'local supervisor*':ab,ti OR 'caregiver'/exp OR 'caregiver*':ab,ti OR 'care-giver*' :ab,ti OR 'peer group'/de OR 'peer education'/de OR (peer NEXT/2 (group* OR support OR relation* OR education)):ab,ti OR 'participatory research'/exp OR 'patient participation'/exp OR 'patient empowerment'/exp OR 'patient engagement'/exp OR 'patient activation'/exp OR 'participatory research':ab,ti OR 'patient involvement':ab,ti OR 'patient participation':ab,ti OR 'patient empowerment':ab,ti OR 'patient activation':ab,ti OR 'patient engagement':ab,ti

OR

(('community'/exp OR 'religion'/exp OR 'church*':ab,ti OR workplace/exp OR 'home'/exp OR 'school'/exp OR 'school teacher'/exp) AND ('patient care'/exp OR 'health care'/exp OR 'disease management'/de OR 'drug therapy'/exp)))

NOT

(('animal'/de OR 'animal experiment'/exp OR 'nonhuman'/de) NOT ('human'/exp OR 'human experiment'/de))

NOT

('juvenile'/exp NOT 'adult'/exp)

NOT

[conference abstract]/lim

**MEDLINE Ovid Search string**

(exp diabetes mellitus/ OR exp Diabetes Complications/ OR exp Blood Glucose Self-Monitoring/ OR exp glycemic control/ OR exp glycemic index/ OR exp glycemic load/ OR exp hyperglycemia/ OR diabet*.ab,ti. OR antidiabet*.ab,ti. OR dm 2.ab,ti. OR dm2.ab,ti. OR dm type 2.ab,ti. OR dm type2.ab,ti. OR niddm.ab,ti. OR t2dm.ab,ti. OR t2 dm.ab,ti. OR ((glycemic OR glycaemic OR glycemia OR glycaemia OR glycemie) ADJ2 (control OR index OR value OR load)).ab,ti. OR hyperglycemi*.ab,ti. OR hyperglycaemi*.ab,ti. OR hyperglucemi*.ab,ti. OR hyper-glycemi*.ab,ti. OR hyper-glycaemi*.ab,ti. OR hyper-glucemi*.ab,ti. OR dysglycemi*.ab,ti. OR ((high OR elevated OR monitor*) ADJ3 (blood glucose OR blood sugar OR plasma glucose OR plasma sugar OR serum glucose OR serum sugar)).ab,ti. OR exp hypertension/ OR Blood Pressure/ OR exp Arterial Pressure/ OR exp Blood Pressure Determination/ OR exp blood pressure monitors/ OR exp Blood Pressure Monitoring, Ambulatory/ OR (hypertens* OR hyper tens* OR blood pressur* OR arterial pressur* OR vascular pressur* OR intravascular pressur* OR blood tension OR arterial tension OR vascular tension OR intravascular tension OR antihypertensive).ab,ti. OR exp insulin resistance/ OR metabolic syndrome*.ab,ti. OR insulin resistance.ab,ti.)

AND

(exp africa south of the sahara/ OR africa south of the sahara.ab,ti. OR sub-sahara* africa*.ab,ti. OR subsahara* africa*.ab,ti. OR black africa*.ab,ti. OR west* africa*.ab,ti. OR east* africa*.ab,ti. OR south* africa*.ab,ti. OR central africa*.ab,ti. OR equatorial africa*.ab,ti. OR middle africa*.ab,ti. OR angola*.ab,ti. OR benin*.ab,ti. OR dahomey.ab,ti. OR botswana*.ab,ti. OR bechuanaland.ab,ti. OR burkina faso.ab,ti. OR burkin*.ab,ti. OR upper volta.ab,ti. OR burundi*.ab,ti. OR urundi.ab,ti. OR cameroon*.ab,ti. OR camerun.ab,ti. OR kamerun.ab,ti. OR cameroun.ab,ti. OR cape verde*.ab,ti. OR cabo verde.ab,ti. OR centrafri*.ab,ti. OR ubangi-shari.ab,ti. OR oubangi-shari.ab,ti. OR chad*.ab,ti. OR tchad.ab,ti. OR comoro*.ab,ti. OR comores.ab,ti. OR comoran.ab,ti. OR comorian.ab,ti. OR congo*.ab,ti. OR kongo.ab,ti. OR zaire.ab,ti. OR cote d`ivoire.ab,ti. OR ivory coast.ab,ti. OR ivorian*.ab,ti. OR djibouti*.ab,ti. OR (afars and issas).ab,ti. OR eritrea*.ab,ti. OR eswatini.ab,ti. OR swazi*.ab,ti. OR ethiopia*.ab,ti. OR abyssinia.ab,ti. OR gabon*.ab,ti. OR gabun.ab,ti. OR gambia*.ab,ti. OR senegambia.ab,ti. OR ghana*.ab,ti. OR gold coast.ab,ti. OR guinea*.ab,ti. OR guinee.ab,ti. OR guine.ab,ti. OR bissau-guinean.ab,ti. OR equatoguinean.ab,ti. OR kenya*.ab,ti. OR lesotho*.ab,ti. OR basutoland.ab,ti. OR liberia*.ab,ti. OR madagasca*.ab,ti. OR malagasy*.ab,ti. OR malawi*.ab,ti. OR nyasaland.ab,ti. OR mali.ab,ti. OR malian*.ab,ti. OR mauritania*.ab,ti. OR mauritanie.ab,ti. OR mauritius.ab,ti. OR republique de maurice.ab,ti. OR mayotte.ab,ti. OR mahoran*.ab,ti. OR mozambi*.ab,ti. OR mocambique.ab,ti. OR namibia*.ab,ti. OR niger*.ab,ti. OR reunion.ab,ti. OR reunionese.ab,ti. OR rwanda*.ab,ti. OR ruanda*.ab,ti. OR senegal*.ab,ti. OR seychell*.ab,ti. OR sierra leon*.ab,ti. OR somali*.ab,ti. OR sudan*.ab,ti. OR tanzania*.ab,ti. OR tansania*.ab,ti. OR tanganyika.ab,ti. OR zanzibar.ab,ti. OR togo*.ab,ti. OR uganda*.ab,ti. OR zambia*.ab,ti. OR sambia*.ab,ti. OR zimbabwe*.ab,ti. OR rhodesia.ab,ti. OR luanda.ab,ti. OR lubango.ab,ti. OR cabinda.ab,ti. OR porto-novo.ab,ti. OR cotonou.ab,ti. OR abomey-calavi.ab,ti. OR gaborone.ab,ti. OR ouagadougou.ab,ti. OR bobo-dioulasso.ab,ti. OR bujumbura.ab,ti. OR yaounde.ab,ti. OR douala.ab,ti. OR praia.ab,ti. OR bangui.ab,ti. OR n`djamena.ab,ti. OR ndjamena.ab,ti. OR moroni.ab,ti. OR brazzaville.ab,ti. OR pointe-noire.ab,ti. OR kinshasa.ab,ti. OR mbuji-mayi.ab,ti. OR lubumbashi.ab,ti. OR kananga.ab,ti. OR kisangani.ab,ti. OR bukavu.ab,ti. OR yamoussoukro.ab,ti. OR abidjan.ab,ti. OR malabo.ab,ti. OR asmara.ab,ti. OR mbabane.ab,ti. OR addis ababa.ab,ti. OR libreville.ab,ti. OR banjul.ab,ti. OR accra.ab,ti. OR kumasi.ab,ti. OR sekondi takoradi.ab,ti. OR conakry.ab,ti. OR bissau.ab,ti. OR nairobi.ab,ti. OR mombasa.ab,ti. OR mombassa.ab,ti. OR maseru.ab,ti. OR monrovia.ab,ti. OR antananarivo.ab,ti. OR lilongwe.ab,ti. OR blantyre-limbe.ab,ti. OR bamako.ab,ti. OR nouakchott.ab,ti. OR port louis.ab,ti. OR maputo.ab,ti. OR matola.ab,ti. OR nampula.ab,ti. OR windhoek.ab,ti. OR niamey.ab,ti. OR abuja.ab,ti. OR lagos.ab,ti. OR kano.ab,ti. OR ibadan.ab,ti. OR port harcourt.ab,ti. OR kigali.ab,ti. OR sao tome*.ab,ti. OR dakar.ab,ti. OR freetown.ab,ti. OR mogadishu.ab,ti. OR hargeisa.ab,ti. OR hargeysa.ab,ti. OR pretoria.ab,ti. OR cape town.ab,ti. OR johannesburg.ab,ti. OR Soweto.ab,ti. OR durban.ab,ti. OR port elizabeth.ab,ti. OR west rand.ab,ti. OR juba.ab,ti. OR khartoum.ab,ti. OR nyala.ab,ti. OR dar es salaam.ab,ti. OR dodoma.ab,ti. OR mwanza.ab,ti. OR lome.ab,ti. OR kampala.ab,ti. OR lusaka.ab,ti. OR harare.ab,ti.)

AND

(exp Telecommunications/ OR exp Telenursing/ OR Telehealth.ab,ti. OR Tele-health.ab,ti. OR Telemedic*.ab,ti. OR Tele-medic*.ab,ti. OR telemetry.ab,ti. OR tele-metry.ab,ti. OR biotelemetry.ab,ti. OR radiotelemetry.ab,ti. OR teleradiometry.ab,ti. OR telesensing.ab,ti. OR tele-sensing.ab,ti. OR telemonitor*.ab,ti. OR tele-monitor*.ab,ti. OR telenurs*.ab,ti. OR tele-nurs*.ab,ti. OR teleconsultat*.ab,ti. OR tele-consultat*.ab,ti. OR telerehabilitation*.ab,ti. OR tele-rehabilitation*.ab,ti. OR tele-ophthalmology.ab,ti. OR teleophthalmology.ab,ti. OR ((virtual OR remote OR distance OR distant OR e) ADJ2 (medic* OR monitor* OR nurs* OR consultat* OR rehabilitation* OR support* OR counsel* OR treatment* OR therap* OR sensing)).ab,ti. OR (emedic* OR emonitor* OR enurs* OR econsultat* OR erehabilitation OR esupport* OR ecounsel* OR etreatment* OR etherap* OR esensing).ab,ti. OR Mhealth.ab,ti. OR M-health.ab,ti. OR mobile health.ab,ti. OR Ehealth.ab,ti. OR E-health.ab,ti. OR exp Distance Counseling/ OR exp Hotlines/ OR internet/ OR exp Internet-Based Intervention/ OR exp Social Media/ OR exp Mobile Applications/ OR e mail*.ab,ti. OR email*.ab,ti. OR (electronic ADJ2 (mail* OR messaging)).ab,ti. OR hotline*.ab,ti. OR interactive voice response system*.ab,ti. OR ((web OR online OR on-line OR internet OR video OR virtual OR tele) ADJ3 (intervention* OR conferenc* OR communication OR seminar* OR information*)).ab,ti. OR internet.ab,ti. OR world wide web.ab,ti. OR www.ab,ti. OR ((mobile OR cell OR smart OR portable) ADJ2 (phone* OR telephone*)).ab,ti. OR social media*.ab,ti. OR facebook.ab,ti. OR twitter.ab,ti. OR telecommunication*.ab,ti. OR broadcasting.ab,ti. OR text messag*.ab,ti. OR texting.ab,ti. OR teleconferenc*.ab,ti. OR videoconferenc*.ab,ti. OR webconferenc*.ab,ti. OR webcast*.ab,ti. OR podcast*.ab,ti. OR webinar*.ab,ti. OR SMS.ab,ti. OR telephone*.ab,ti. OR smartphone*.ab,ti. OR ((mobile OR portable OR tablet) ADJ2 (app OR apps OR application*)).ab,ti. OR website.ab,ti. OR homepage.ab,ti. OR mobile technology.ab,ti. OR exp ambulatory Care/ OR decentrali*.ab,ti. OR ((ambulatory OR dispensary OR extramural OR extra-mural OR outpatient OR outpatient health* OR out-patient OR out-patient health* OR out-of-office) ADJ2 (care OR healthcare OR service* OR setting* OR monitoring* OR treatment* OR therap*)).ab,ti. OR Community Health Services/ OR exp Community Health Nursing/ OR exp Community Pharmacy Services/ OR exp Community Integration/ OR exp Uncompensated Care/ OR exp community medicine/ OR rehabilitation/ OR ((community OR community health* OR district OR public health OR nurse-led OR pharmacist-led OR nurse-based OR pharmacist-based OR social service OR collaborative) ADJ3 (care OR healthcare OR nurs* OR integration OR service* OR program* OR medicine OR support* OR engagement OR intervention* OR deliver* OR outreach OR distribution OR network* OR participatory method* OR rehabilitation* OR resource* OR strateg* OR project* OR approach* OR testing OR screening*)).ab,ti. OR task shar*.ab,ti. OR task shift*.ab,ti. OR exp Home Care Services/ OR exp Adult Day Care Centers/ OR ((adult OR home OR domicil* OR domestic OR visiting OR residential OR residence) ADJ3 (care OR healthcare OR nurs* OR help OR assistance OR service* OR treatment* OR therap* OR medication review* OR pharmac* OR monitor* OR visit* OR agenc* OR testing OR screening* OR diagnos*)).ab,ti. OR homecare.ab,ti. OR house call*.ab,ti. OR exp Diagnostic Tests, Routine/ OR housebound patient*.ab,ti. OR adult day care.ab,ti. OR family service*.ab,ti. OR family involve*.ab,ti. OR family based.ab,ti. OR family-based.ab,ti. OR family health*.ab,ti. OR exp Ambulatory Care/ OR out-of-facilit*.ab,ti. OR exp Community Health Centers/ OR ((health OR health service OR sanitary) ADJ2 (center* OR centre* OR clinic* OR institute* OR unit* OR resort* OR facility OR facilities)).ab,ti. OR exp Pharmacies/ OR exp Pharmaceutical Services, Online/ OR apothecary.ab,ti. OR chemist shop*.ab,ti. OR chemist`s shop*.ab,ti. OR pharmacies.ab,ti. OR pharmacy.ab,ti. OR pharmaceutical service*.ab,ti. OR exp Rural Health Services/ OR School Health Services/ OR exp School Nursing/ OR ((rural OR school* OR workplace OR worksite OR work-place OR work-site) ADJ3 (health* OR care OR medicine OR nurse OR nurses OR nursing OR intervention* OR program* OR approach* OR service* OR strateg*)).ab,ti. OR exp self care/ OR (self ADJ2 (care* OR help OR management OR monitoring OR treatment* OR medication OR nurturance OR testing OR measur*)).ab,ti. OR selfcare.ab,ti. OR selfhelp.ab,ti. OR selfmanagement.ab,ti. OR selfmonitor*.ab,ti. OR selftreatment.ab,ti. OR selfmedication*.ab,ti. OR exp social network/ OR exp social care/ OR ((social OR spiritual) ADJ2 (care OR support OR network* OR service* OR work)).ab,ti. OR exp Self-Help Groups/ OR support group*.ab,ti. OR support program*.ab,ti. OR paramedical.ab,ti. OR para-medical.ab,ti. OR non-health.ab,ti. OR non-healthcare.ab,ti. OR non-medical.ab,ti. OR ((health OR healthcare) ADJ2 (support worker* OR assistant* OR aid*)).ab,ti. OR ((community OR district OR public health OR assistan* OR aid* OR staff* OR manpower OR personnel) ADJ4 (nurse OR nurses OR nursing OR matron*)).ab,ti. OR midlevel health professional*.ab,ti. OR midlevel health provider*.ab,ti. OR Allied Health Personnel/ OR exp Pharmacy Technicians/ OR exp Community Health Workers/ OR exp Home Health Aides/ OR exp Licensed Practical Nurses/ OR exp nurses/ OR exp Nursing Assistants/ OR exp nursing staff/ OR exp pharmacists/ OR ((community OR community based OR village OR lay OR allied health OR auxiliary health OR health auxiliary) ADJ3 (worker* OR provider* OR personnel OR profession* OR staff OR aide* OR supporter* OR volunteer* OR advocate* OR team* OR group* OR pharmacist*)).ab,ti. OR barefoot doctor*.ab,ti. OR local supervisor*.ab,ti. OR exp Caregivers/ OR caregiver*.ab,ti. OR care giver*.ab,ti. OR exp peer group/ OR (peer ADJ2 (group* OR support OR relation* OR education)).ab,ti. OR exp Community-Based Participatory Research/ OR exp Patient Participation/ OR participatory research.ab,ti. OR patient involvement.ab,ti. OR patient participation.ab,ti. OR patient empowerment.ab,ti. OR patient activation.ab,ti. OR patient engagement.ab,ti. OR ((exp community networks/ OR exp religion/ OR church*.ab,ti. OR exp workplace/ OR exp schools/ OR exp school teachers/) AND (exp patient care/ OR disease management/ OR exp drug therapy/)))

NOT

((exp animals/ OR exp Animal Experimentation/) NOT humans/)

NOT

((exp infant/ OR exp child/ OR adolescent/) NOT exp adult/)

**CINAHL EBSCOhost Search string**

((MH "diabetes mellitus+") OR (MH "Diabetic Patients") OR (MH "Diabetes Education") OR (MH "Diabetes Educators") OR (MH "Blood Glucose Monitoring+") OR (MH "glycemic control") OR (MH "glycemic index") OR (MH "glycemic load") OR (MH "hyperglycemia+") OR (TI diabet* OR AB diabet*) OR (TI antidiabet* OR AB antidiabet*) OR (TI "dm 2" OR AB "dm 2") OR (TI dm2 OR AB dm2) OR (TI "dm type 2" OR AB "dm type 2") OR (TI "dm type2" OR AB "dm type2") OR (TI niddm OR AB niddm) OR (TI t2dm OR AB t2dm) OR (TI "t2 dm" OR AB "t2 dm") OR (((TI glycemic OR AB glycemic) OR (TI glycaemic OR AB glycaemic) OR (TI glycemia OR AB glycemia) OR (TI glycaemia OR AB glycaemia) OR (TI glycemie OR AB glycemie)) N2 ((TI control OR AB control) OR (TI index OR AB index) OR (TI value OR AB value) OR (TI load OR AB load))) OR (TI hyperglycemi* OR AB hyperglycemi*) OR (TI hyperglycaemi* OR AB hyperglycaemi*) OR (TI hyperglucemi* OR AB hyperglucemi*) OR (TI hyper-glycemi* OR AB hyper-glycemi*) OR (TI hyper-glycaemi* OR AB hyper-glycaemi*) OR (TI hyper-glucemi* OR AB hyper-glucemi*) OR (TI dysglycemi* OR AB dysglycemi*) OR (((TI high OR AB high) OR (TI elevated OR AB elevated) OR (TI monitor* OR AB monitor*)) N3 ((TI "blood glucose" OR AB "blood glucose") OR (TI "blood sugar" OR AB "blood sugar") OR (TI "plasma glucose" OR AB "plasma glucose") OR (TI "plasma sugar" OR AB "plasma sugar") OR (TI "serum glucose" OR AB "serum glucose") OR (TI "serum sugar" OR AB "serum sugar"))) OR (MH "hypertension+") OR (MH "Blood Pressure") OR (MH "Arterial Pressure") OR (MH "Blood Pressure Determination") OR (MH "blood pressure devices+") OR (MH "Blood Pressure Monitoring, Ambulatory") OR ((TI hypertens* OR AB hypertens*) OR (TI "hyper tens*" OR AB "hyper tens*") OR (TI "blood pressur*" OR AB "blood pressur*") OR (TI "arterial pressur*" OR AB "arterial pressur*") OR (TI "vascular pressur*" OR AB "vascular pressur*") OR (TI "intravascular pressur*" OR AB "intravascular pressur*") OR (TI "blood tension" OR AB "blood tension") OR (TI "arterial tension" OR AB "arterial tension") OR (TI "vascular tension" OR AB "vascular tension") OR (TI "intravascular tension" OR AB "intravascular tension") OR (TI antihypertensive OR AB antihypertensive)) OR (MH "insulin resistance+") OR (TI "metabolic syndrome*" OR AB "metabolic syndrome*") OR (TI "insulin resistance" OR AB "insulin resistance"))

AND

((MH "africa south of the sahara+") OR (TI "africa south of the sahara" OR AB "africa south of the sahara") OR (TI "sub-sahara* africa*" OR AB "sub-sahara* africa*") OR (TI "subsahara* africa*" OR AB "subsahara* africa*") OR (TI "black africa*" OR AB "black africa*") OR (MH "Africa, Western+") OR (TI "west* africa*" OR AB "west* africa*") OR (TI "east* africa*" OR AB "east* africa*") OR (TI "south* africa*" OR AB "south* africa*") OR (TI "central africa*" OR AB "central africa*") OR (TI "equatorial africa*" OR AB "equatorial africa*") OR (TI "middle africa*" OR AB "middle africa*") OR (TI angola* OR AB angola*) OR (TI benin* OR AB benin*) OR (TI dahomey OR AB dahomey) OR (TI botswana* OR AB botswana*) OR (TI bechuanaland OR AB bechuanaland) OR (TI "burkina faso" OR AB "burkina faso") OR (TI burkin* OR AB burkin*) OR (TI "upper volta" OR AB "upper volta") OR (TI burundi* OR AB burundi*) OR (TI urundi OR AB urundi) OR (TI cameroon* OR AB cameroon*) OR (TI camerun OR AB camerun) OR (TI kamerun OR AB kamerun) OR (TI cameroun OR AB cameroun) OR (TI "cape verde*" OR AB "cape verde*") OR (TI "cabo verde" OR AB "cabo verde") OR (TI centrafri* OR AB centrafri*) OR (TI ubangi-shari OR AB ubangi-shari) OR (TI oubangi-shari OR AB oubangi-shari) OR (TI chad* OR AB chad*) OR (TI tchad OR AB tchad) OR (TI comoro* OR AB comoro*) OR (TI comores OR AB comores) OR (TI comoran OR AB comoran) OR (TI comorian OR AB comorian) OR (TI congo* OR AB congo*) OR (TI kongo OR AB kongo) OR (TI zaire OR AB zaire) OR (TI "cote d`ivoire" OR AB "cote d`ivoire") OR (TI "ivory coast" OR AB "ivory coast") OR (TI ivorian* OR AB ivorian*) OR (TI djibouti* OR AB djibouti*) OR (TI "afars and issas" OR AB "afars and issas") OR (TI eritrea* OR AB eritrea*) OR (TI eswatini OR AB eswatini) OR (TI swazi* OR AB swazi*) OR (TI ethiopia* OR AB ethiopia*) OR (TI abyssinia OR AB abyssinia) OR (TI gabon* OR AB gabon*) OR (TI gabun OR AB gabun) OR (TI gambia* OR AB gambia*) OR (TI senegambia OR AB senegambia) OR (TI ghana* OR AB ghana*) OR (TI "gold coast" OR AB "gold coast") OR (TI guinea* OR AB guinea*) OR (TI guinee OR AB guinee) OR (TI guine OR AB guine) OR (TI bissau-guinean OR AB bissau-guinean) OR (TI equatoguinean OR AB equatoguinean) OR (TI kenya* OR AB kenya*) OR (TI lesotho* OR AB lesotho*) OR (TI basutoland OR AB basutoland) OR (TI liberia* OR AB liberia*) OR (TI madagasca* OR AB madagasca*) OR (TI malagasy* OR AB malagasy*) OR (TI malawi* OR AB malawi*) OR (TI nyasaland OR AB nyasaland) OR (TI mali OR AB mali) OR (TI malian* OR AB malian*) OR (TI mauritania* OR AB mauritania*) OR (TI mauritanie OR AB mauritanie) OR (TI mauritius OR AB mauritius) OR (TI "republique de maurice" OR AB "republique de maurice") OR (TI mayotte OR AB mayotte) OR (TI mahoran* OR AB mahoran*) OR (TI mozambi* OR AB mozambi*) OR (TI mocambique OR AB mocambique) OR (TI namibia* OR AB namibia*) OR (TI niger* OR AB niger*) OR (TI reunion OR AB reunion) OR (TI reunionese OR AB reunionese) OR (TI rwanda* OR AB rwanda*) OR (TI ruanda* OR AB ruanda*) OR (TI senegal* OR AB senegal*) OR (TI seychell* OR AB seychell*) OR (TI "sierra leon*" OR AB "sierra leon*") OR (TI somali* OR AB somali*) OR (TI sudan* OR AB sudan*) OR (TI tanzania* OR AB tanzania*) OR (TI tansania* OR AB tansania*) OR (TI tanganyika OR AB tanganyika) OR (TI zanzibar OR AB zanzibar) OR (TI togo* OR AB togo*) OR (TI uganda* OR AB uganda*) OR (TI zambia* OR AB zambia*) OR (TI sambia* OR AB sambia*) OR (TI zimbabwe* OR AB zimbabwe*) OR (TI rhodesia OR AB rhodesia) OR (TI luanda OR AB luanda) OR (TI lubango OR AB lubango) OR (TI cabinda OR AB cabinda) OR (TI porto-novo OR AB porto-novo) OR (TI cotonou OR AB cotonou) OR (TI abomey-calavi OR AB abomey-calavi) OR (TI gaborone OR AB gaborone) OR (TI ouagadougou OR AB ouagadougou) OR (TI bobo-dioulasso OR AB bobo-dioulasso) OR (TI bujumbura OR AB bujumbura) OR (TI yaounde OR AB yaounde) OR (TI douala OR AB douala) OR (TI praia OR AB praia) OR (TI bangui OR AB bangui) OR (TI n`djamena OR AB n`djamena) OR (TI ndjamena OR AB ndjamena) OR (TI moroni OR AB moroni) OR (TI brazzaville OR AB brazzaville) OR (TI pointe-noire OR AB pointe-noire) OR (TI kinshasa OR AB kinshasa) OR (TI mbuji-mayi OR AB mbuji-mayi) OR (TI lubumbashi OR AB lubumbashi) OR (TI kananga OR AB kananga) OR (TI kisangani OR AB kisangani) OR (TI bukavu OR AB bukavu) OR (TI yamoussoukro OR AB yamoussoukro) OR (TI abidjan OR AB abidjan) OR (TI malabo OR AB malabo) OR (TI asmara OR AB asmara) OR (TI mbabane OR AB mbabane) OR (TI "addis ababa" OR AB "addis ababa") OR (TI libreville OR AB libreville) OR (TI banjul OR AB banjul) OR (TI accra OR AB accra) OR (TI kumasi OR AB kumasi) OR (TI "sekondi takoradi" OR AB "sekondi takoradi") OR (TI conakry OR AB conakry) OR (TI bissau OR AB bissau) OR (TI nairobi OR AB nairobi) OR (TI mombasa OR AB mombasa) OR (TI mombassa OR AB mombassa) OR (TI maseru OR AB maseru) OR (TI monrovia OR AB monrovia) OR (TI antananarivo OR AB antananarivo) OR (TI lilongwe OR AB lilongwe) OR (TI blantyre-limbe OR AB blantyre-limbe) OR (TI bamako OR AB bamako) OR (TI nouakchott OR AB nouakchott) OR (TI "port louis" OR AB "port louis") OR (TI maputo OR AB maputo) OR (TI matola OR AB matola) OR (TI nampula OR AB nampula) OR (TI windhoek OR AB windhoek) OR (TI niamey OR AB niamey) OR (TI abuja OR AB abuja) OR (TI lagos OR AB lagos) OR (TI kano OR AB kano) OR (TI ibadan OR AB ibadan) OR (TI "port harcourt" OR AB "port harcourt") OR (TI kigali OR AB kigali) OR (TI "sao tome*" OR AB "sao tome*") OR (TI dakar OR AB dakar) OR (TI freetown OR AB freetown) OR (TI mogadishu OR AB mogadishu) OR (TI hargeisa OR AB hargeisa) OR (TI hargeysa OR AB hargeysa) OR (TI pretoria OR AB pretoria) OR (TI "cape town" OR AB "cape town") OR (TI johannesburg OR AB johannesburg) OR (TI Soweto OR AB Soweto) OR (TI durban OR AB durban) OR (TI "port elizabeth" OR AB "port elizabeth") OR (TI "west rand" OR AB "west rand") OR (TI juba OR AB juba) OR (TI khartoum OR AB khartoum) OR (TI nyala OR AB nyala) OR (TI "dar es salaam" OR AB "dar es salaam") OR (TI dodoma OR AB dodoma) OR (TI mwanza OR AB mwanza) OR (TI lome OR AB lome) OR (TI kampala OR AB kampala) OR (TI lusaka OR AB lusaka) OR (TI harare OR AB harare))

AND

((MH "Decentralization") OR (MH "Telecommunications+") OR (MH "Telemetry") OR (TI Telehealth OR AB Telehealth) OR (TI Tele-health OR AB Tele-health) OR (TI Telemedic* OR AB Telemedic*) OR (TI Tele-medic* OR AB Tele-medic*) OR (TI telemetry OR AB telemetry) OR (TI tele-metry OR AB tele-metry) OR (TI biotelemetry OR AB biotelemetry) OR (TI radiotelemetry OR AB radiotelemetry) OR (TI teleradiometry OR AB teleradiometry) OR (TI telesensing OR AB telesensing) OR (TI tele-sensing OR AB tele-sensing) OR (TI telemonitor* OR AB telemonitor*) OR (TI tele-monitor* OR AB tele-monitor*) OR (TI telenurs* OR AB telenurs*) OR (TI tele-nurs* OR AB tele-nurs*) OR (TI teleconsultat* OR AB teleconsultat*) OR (TI tele-consultat* OR AB tele-consultat*) OR (TI telerehabilitation* OR AB telerehabilitation*) OR (TI tele-rehabilitation* OR AB tele-rehabilitation*) OR (TI tele-ophthalmology OR AB tele-ophthalmology) OR (TI teleophthalmology OR AB teleophthalmology) OR (((TI virtual OR AB virtual) OR (TI remote OR AB remote) OR (TI distance OR AB distance) OR (TI distant OR AB distant) OR (TI e OR AB e)) N2 ((TI medic* OR AB medic*) OR (TI monitor* OR AB monitor*) OR (TI nurs* OR AB nurs*) OR (TI consultat* OR AB consultat*) OR (TI rehabilitation* OR AB rehabilitation*) OR (TI support* OR AB support*) OR (TI counsel* OR AB counsel*) OR (TI treatment* OR AB treatment*) OR (TI therap* OR AB therap*) OR (TI sensing OR AB sensing))) OR (TI emedic* OR AB emedic*) OR (TI emonitor* OR AB emonitor*) OR (TI enurs* OR AB enurs*) OR (TI econsultat* OR AB econsultat*) OR (TI erehabilitation OR AB erehabilitation) OR (TI esupport* OR AB esupport*) OR (TI ecounsel* OR AB ecounsel*) OR (TI etreatment* OR AB etreatment*) OR (TI etherap* OR AB etherap*) OR (TI esensing OR AB esensing) OR (TI Mhealth OR AB Mhealth) OR (TI M-health OR AB M-health) OR (TI "mobile health" OR AB "mobile health") OR (TI Ehealth OR AB Ehealth) OR (TI E-health OR AB E-health) OR (MH "Telephone Information Services") OR (MH "Mobile Applications") OR (TI "e mail*" OR AB "e mail*") OR (TI email* OR AB email*) OR ((TI electronic OR AB electronic) N2 ((TI mail* OR AB mail*) OR (TI messaging OR AB messaging))) OR (TI hotline* OR AB hotline*) OR (TI "interactive voice response system*" OR AB "interactive voice response system*") OR (((TI web OR AB web) OR (TI online OR AB online) OR (TI on-line OR AB on-line) OR (TI internet OR AB internet) OR (TI video OR AB video) OR (TI virtual OR AB virtual) OR (TI tele OR AB tele)) N3 ((TI intervention* OR AB intervention*) OR (TI conferenc* OR AB conferenc*) OR (TI communication OR AB communication) OR (TI seminar* OR AB seminar*) OR (TI information* OR AB information*))) OR (TI internet OR AB internet) OR (TI "world wide web" OR AB "world wide web") OR (TI www OR AB www) OR (((TI mobile OR AB mobile) OR (TI cell OR AB cell) OR (TI smart OR AB smart) OR (TI portable OR AB portable)) N2 ((TI phone* OR AB phone*) OR (TI telephone* OR AB telephone*))) OR (TI "social media*" OR AB "social media*") OR (TI facebook OR AB facebook) OR (TI twitter OR AB twitter) OR (TI telecommunication* OR AB telecommunication*) OR (TI broadcasting OR AB broadcasting) OR (TI "text messag*" OR AB "text messag*") OR (TI texting OR AB texting) OR (TI teleconferenc* OR AB teleconferenc*) OR (TI videoconferenc* OR AB videoconferenc*) OR (TI webconferenc* OR AB webconferenc*) OR (TI webcast* OR AB webcast*) OR (TI podcast* OR AB podcast*) OR (TI webinar* OR AB webinar*) OR (TI SMS OR AB SMS) OR (TI telephone* OR AB telephone*) OR (TI smartphone* OR AB smartphone*) OR (((TI mobile OR AB mobile) OR (TI portable OR AB portable) OR (TI tablet OR AB tablet)) N2 ((TI app OR AB app) OR (TI apps OR AB apps) OR (TI application* OR AB application*))) OR (TI website OR AB website) OR (TI homepage OR AB homepage) OR (TI "mobile technology" OR AB "mobile technology") OR (MH "ambulatory Care") OR (MH "ambulatory Care Information Systems") OR (MH "ambulatory Care Facilities") OR (MH "ambulatory Care Nursing") OR (TI decentrali* OR AB decentrali*) OR (((TI ambulatory OR AB ambulatory) OR (TI dispensary OR AB dispensary) OR (TI extramural OR AB extramural) OR (TI extra-mural OR AB extra-mural) OR (TI outpatient OR AB outpatient) OR (TI "outpatient health*" OR AB "outpatient health*") OR (TI out-patient OR AB out-patient) OR (TI "out-patient health*" OR AB "out-patient health*") OR (TI out-of-office OR AB out-of-office)) N2 ((TI care OR AB care) OR (TI healthcare OR AB healthcare) OR (TI service* OR AB service*) OR (TI setting* OR AB setting*) OR (TI monitoring* OR AB monitoring*) OR (TI treatment* OR AB treatment*) OR (TI therap* OR AB therap*))) OR (MH "Community Health Services+") OR (MH "community medicine") OR (MH "rehabilitation") OR (MH "Community Programs") OR (MH "Uncompensated Care") OR (((TI community OR AB community) OR (TI "community health*" OR AB "community health*") OR (TI district OR AB district) OR (TI "public health" OR AB "public health") OR (TI nurse-led OR AB nurse-led) OR (TI pharmacist-led OR AB pharmacist-led) OR (TI nurse-based OR AB nurse-based) OR (TI pharmacist-based OR AB pharmacist-based) OR (TI "social service" OR AB "social service") OR (TI collaborative OR AB collaborative)) N3 ((TI care OR AB care) OR (TI healthcare OR AB healthcare) OR (TI nurs* OR AB nurs*) OR (TI integration OR AB integration) OR (TI service* OR AB service*) OR (TI program* OR AB program*) OR (TI medicine OR AB medicine) OR (TI support* OR AB support*) OR (TI engagement OR AB engagement) OR (TI intervention* OR AB intervention*) OR (TI deliver* OR AB deliver*) OR (TI outreach OR AB outreach) OR (TI distribution OR AB distribution) OR (TI network* OR AB network*) OR (TI "participatory method*" OR AB "participatory method*") OR (TI rehabilitation* OR AB rehabilitation*) OR (TI resource* OR AB resource*) OR (TI strateg* OR AB strateg*) OR (TI project* OR AB project*) OR (TI approach* OR AB approach*) OR (TI testing OR AB testing) OR (TI screening* OR AB screening*))) OR (TI "task shar*" OR AB "task shar*") OR (TI "task shift*" OR AB "task shift*") OR (MH "Adult Day Center (Saba CCC)") OR (MH "Residential Care+") OR (((TI adult OR AB adult) OR (TI home OR AB home) OR (TI domicil* OR AB domicil*) OR (TI domestic OR AB domestic) OR (TI visiting OR AB visiting) OR (TI residential OR AB residential) OR (TI residence OR AB residence)) N3 ((TI care OR AB care) OR (TI healthcare OR AB healthcare) OR (TI nurs* OR AB nurs*) OR (TI help OR AB help) OR (TI assistance OR AB assistance) OR (TI service* OR AB service*) OR (TI treatment* OR AB treatment*) OR (TI therap* OR AB therap*) OR (TI "medication review*" OR AB "medication review*") OR (TI pharmac* OR AB pharmac*) OR (TI monitor* OR AB monitor*) OR (TI visit* OR AB visit*) OR (TI agenc* OR AB agenc*) OR (TI testing OR AB testing) OR (TI screening* OR AB screening*) OR (TI diagnos* OR AB diagnos*))) OR (TI homecare OR AB homecare) OR (TI "house call*" OR AB "house call*") OR (MH "Diagnostic Tests, Routine") OR (TI "housebound patient*" OR AB "housebound patient*") OR (TI "adult day care" OR AB "adult day care") OR (TI "family service*" OR AB "family service*") OR (TI "family involve*" OR AB "family involve*") OR (TI "family based" OR AB "family based") OR (TI family-based OR AB family-based) OR (TI "family health*" OR AB "family health*") OR (TI out-of-facilit* OR AB out-of-facilit*) OR (MH "Community Health Centers+") OR (((TI health OR AB health) OR (TI "health service" OR AB "health service") OR (TI sanitary OR AB sanitary)) N2 ((TI center* OR AB center*) OR (TI centre* OR AB centre*) OR (TI clinic* OR AB clinic*) OR (TI institute* OR AB institute*) OR (TI unit* OR AB unit*) OR (TI resort* OR AB resort*) OR (TI facility OR AB facility) OR (TI facilities OR AB facilities))) OR (MH "Pharmacy, Retail") OR (TI apothecary OR AB apothecary) OR (TI "chemist shop*" OR AB "chemist shop*") OR (TI "chemist`s shop*" OR AB "chemist`s shop*") OR (TI pharmacies OR AB pharmacies) OR (TI pharmacy OR AB pharmacy) OR (TI "pharmaceutical service*" OR AB "pharmaceutical service*") OR (MH "Rural Health Services") OR (MH "Rural Health Centers") OR (MH "Rural Health Personnel") OR (MH "School Health Services+") OR (((TI rural OR AB rural) OR (TI school* OR AB school*) OR (TI workplace OR AB workplace) OR (TI worksite OR AB worksite) OR (TI work-place OR AB work-place) OR (TI work-site OR AB work-site)) N3 ((TI health* OR AB health*) OR (TI care OR AB care) OR (TI medicine OR AB medicine) OR (TI nurse OR AB nurse) OR (TI nurses OR AB nurses) OR (TI nursing OR AB nursing) OR (TI intervention* OR AB intervention*) OR (TI program* OR AB program*) OR (TI approach* OR AB approach*) OR (TI service* OR AB service*) OR (TI strateg* OR AB strateg*))) OR (MH "self care+") OR ((TI self OR AB self) N2 ((TI care* OR AB care*) OR (TI help OR AB help) OR (TI management OR AB management) OR (TI monitoring OR AB monitoring) OR (TI treatment* OR AB treatment*) OR (TI medication OR AB medication) OR (TI nurturance OR AB nurturance) OR (TI testing OR AB testing) OR (TI measur* OR AB measur*))) OR (TI selfcare OR AB selfcare) OR (TI selfhelp OR AB selfhelp) OR (TI selfmanagement OR AB selfmanagement) OR (TI selfmonitor* OR AB selfmonitor*) OR (TI selftreatment OR AB selftreatment) OR (TI selfmedication* OR AB selfmedication*) OR (MH "social networks") OR (((TI social OR AB social) OR (TI spiritual OR AB spiritual)) N2 ((TI care OR AB care) OR (TI support OR AB support) OR (TI network* OR AB network*) OR (TI service* OR AB service*) OR (TI work OR AB work))) OR (MH "Support Groups+") OR (TI "support group*" OR AB "support group*") OR (TI "support program*" OR AB "support program*") OR (TI paramedical OR AB paramedical) OR (TI para-medical OR AB para-medical) OR (TI non-health OR AB non-health) OR (TI non-healthcare OR AB non-healthcare) OR (TI non-medical OR AB non-medical) OR ((health OR healthcare) N2 ("support worker*" OR assistant* OR aid*)) OR (((TI community OR AB community) OR (TI district OR AB district) OR (TI "public health" OR AB "public health") OR (TI assistan* OR AB assistan*) OR (TI aid* OR AB aid*) OR (TI staff* OR AB staff*) OR (TI manpower OR AB manpower) OR (TI personnel OR AB personnel)) N4 ((TI nurse OR AB nurse) OR (TI nurses OR AB nurses) OR (TI nursing OR AB nursing) OR (TI matron* OR AB matron*))) OR (TI "midlevel health professional*" OR AB "midlevel health professional*") OR (TI "midlevel health provider*" OR AB "midlevel health provider*") OR (MH "Allied Health Personnel") OR (MH "Pharmacy Technicians") OR (MH "Community Health Workers+") OR (MH "Home Health Aides") OR (MH "nurses+") OR (MH "Nursing Assistants") OR (MH "pharmacists") OR (MH "Allied Health Professions") OR (((TI community OR AB community) OR (TI "community based" OR AB "community based") OR (TI village OR AB village) OR (TI lay OR AB lay) OR (TI "allied health" OR AB "allied health") OR (TI "auxiliary health" OR AB "auxiliary health") OR (TI "health auxiliary" OR AB "health auxiliary")) N3 ((TI worker* OR AB worker*) OR (TI provider* OR AB provider*) OR (TI personnel OR AB personnel) OR (TI profession* OR AB profession*) OR (TI staff OR AB staff) OR (TI aide* OR AB aide*) OR (TI supporter* OR AB supporter*) OR (TI volunteer* OR AB volunteer*) OR (TI advocate* OR AB advocate*) OR (TI team* OR AB team*) OR (TI group* OR AB group*) OR (TI pharmacist* OR AB pharmacist*))) OR (TI "barefoot doctor*" OR AB "barefoot doctor*") OR (TI "local supervisor*" OR AB "local supervisor*") OR (MH "Caregivers") OR (TI caregiver* OR AB caregiver*) OR (TI "care giver*" OR AB "care giver*") OR (MH "peer group") OR ((TI peer OR AB peer) N2 ((TI group* OR AB group*) OR (TI support OR AB support) OR (TI relation* OR AB relation*) OR (TI education OR AB education))) OR (MH "Community-Based Participatory Research+") OR (MH "Patient Participation+") OR (TI "participatory research" OR AB "participatory research") OR (TI "patient involvement" OR AB "patient involvement") OR (TI "patient participation" OR AB "patient participation") OR (TI "patient empowerment" OR AB "patient empowerment") OR (TI "patient activation" OR AB "patient activation") OR (TI "patient engagement" OR AB "patient engagement") OR (((MH "community networks") OR (MH "churches") OR (TI church* OR AB church*) OR (MH "work environment+") OR (MH "Home Environment") OR (MH "schools+") OR (MH "teachers")) AND ((MH "patient care+") OR (MH "drug therapy+"))))

**Scopus Search string**

Scopus cannot correctly process a search, if several terms in a bracket each have field codes. The field codes need to be pooled out, i.e. the field code written only once for several terms with the aid of brackets.

(TITLE-ABS("diabet*") OR TITLE-ABS("antidiabet*") OR TITLE-ABS("dm 2") OR TITLE-ABS("dm2") OR TITLE-ABS("dm type 2") OR TITLE-ABS("dm type2") OR TITLE-ABS("niddm") OR TITLE-ABS("t2dm") OR TITLE-ABS("t2 dm") OR ((TITLE-ABS("glycemic" OR "glycaemic" OR "glycemia" OR "glycaemia" OR "glycemie")) W/2 (TITLE-ABS("control" OR "index" OR "value" OR "load"))) OR TITLE-ABS("hyperglycemi*") OR TITLE-ABS("hyperglycaemi*") OR TITLE-ABS("hyperglucemi*") OR TITLE-ABS("hyper-glycemi*") OR TITLE-ABS("hyper-glycaemi*") OR TITLE-ABS("hyper-glucemi*") OR TITLE-ABS("dysglycemi*") OR ((TITLE-ABS("high" OR "elevated" OR "monitor*")) W/3 (TITLE-ABS("blood glucose" OR "blood sugar" OR "plasma glucose" OR "plasma sugar" OR "serum glucose" OR "serum sugar"))) OR (TITLE-ABS("hypertens*") OR TITLE-ABS("hyper tens*") OR TITLE-ABS("blood pressur*") OR TITLE-ABS("arterial pressur*") OR TITLE-ABS("vascular pressur*") OR TITLE-ABS("intravascular pressur*") OR TITLE-ABS("blood tension") OR TITLE-ABS("arterial tension") OR TITLE-ABS("vascular tension") OR TITLE-ABS("intravascular tension") OR TITLE-ABS("antihypertensive")) OR TITLE-ABS("metabolic syndrome*") OR TITLE-ABS("insulin resistance"))

AND

(TITLE-ABS("africa south of the sahara") OR TITLE-ABS("sub-sahara* africa*") OR TITLE-ABS("subsahara* africa*") OR TITLE-ABS("black africa*") OR TITLE-ABS("west* africa*") OR TITLE-ABS("east* africa*") OR TITLE-ABS("south* africa*") OR TITLE-ABS("central africa*") OR TITLE-ABS("equatorial africa*") OR TITLE-ABS("middle africa*") OR TITLE-ABS("angola*") OR TITLE-ABS("benin*") OR TITLE-ABS("dahomey") OR TITLE-ABS("botswana*") OR TITLE-ABS("bechuanaland") OR TITLE-ABS("burkina faso") OR TITLE-ABS("burkin*") OR TITLE-ABS("upper volta") OR TITLE-ABS("burundi*") OR TITLE-ABS("urundi") OR TITLE-ABS("cameroon*") OR TITLE-ABS("camerun") OR TITLE-ABS("kamerun") OR TITLE-ABS("cameroun") OR TITLE-ABS("cape verde*") OR TITLE-ABS("cabo verde") OR TITLE-ABS("centrafri*") OR TITLE-ABS("ubangi-shari") OR TITLE-ABS("oubangi-shari") OR TITLE-ABS("chad*") OR TITLE-ABS("tchad") OR TITLE-ABS("comoro*") OR TITLE-ABS("comores") OR TITLE-ABS("comoran") OR TITLE-ABS("comorian") OR TITLE-ABS("congo*") OR TITLE-ABS("kongo") OR TITLE-ABS("zaire") OR TITLE-ABS("cote d`ivoire") OR TITLE-ABS("ivory coast") OR TITLE-ABS("ivorian*") OR TITLE-ABS("djibouti*") OR TITLE-ABS("afars and issas") OR TITLE-ABS("eritrea*") OR TITLE-ABS("eswatini") OR TITLE-ABS("swazi*") OR TITLE-ABS("ethiopia*") OR TITLE-ABS("abyssinia") OR TITLE-ABS("gabon*") OR TITLE-ABS("gabun") OR TITLE-ABS("gambia*") OR TITLE-ABS("senegambia") OR TITLE-ABS("ghana*") OR TITLE-ABS("gold coast") OR TITLE-ABS("guinea*") OR TITLE-ABS("guinee") OR TITLE-ABS("guine") OR TITLE-ABS("bissau-guinean") OR TITLE-ABS("equatoguinean") OR TITLE-ABS("kenya*") OR TITLE-ABS("lesotho*") OR TITLE-ABS("basutoland") OR TITLE-ABS("liberia*") OR TITLE-ABS("madagasca*") OR TITLE-ABS("malagasy*") OR TITLE-ABS("malawi*") OR TITLE-ABS("nyasaland") OR TITLE-ABS("mali") OR TITLE-ABS("malian*") OR TITLE-ABS("mauritania*") OR TITLE-ABS("mauritanie") OR TITLE-ABS("mauritius") OR TITLE-ABS("republique de maurice") OR TITLE-ABS("mayotte") OR TITLE-ABS("mahoran*") OR TITLE-ABS("mozambi*") OR TITLE-ABS("mocambique") OR TITLE-ABS("namibia*") OR TITLE-ABS("niger*") OR TITLE-ABS("reunion") OR TITLE-ABS("reunionese") OR TITLE-ABS("rwanda*") OR TITLE-ABS("ruanda*") OR TITLE-ABS("senegal*") OR TITLE-ABS("seychell*") OR TITLE-ABS("sierra leon*") OR TITLE-ABS("somali*") OR TITLE-ABS("sudan*") OR TITLE-ABS("tanzania*") OR TITLE-ABS("tansania*") OR TITLE-ABS("tanganyika") OR TITLE-ABS("zanzibar") OR TITLE-ABS("togo*") OR TITLE-ABS("uganda*") OR TITLE-ABS("zambia*") OR TITLE-ABS("sambia*") OR TITLE-ABS("zimbabwe*") OR TITLE-ABS("rhodesia") OR TITLE-ABS("luanda") OR TITLE-ABS("lubango") OR TITLE-ABS("cabinda") OR TITLE-ABS("porto-novo") OR TITLE-ABS("cotonou") OR TITLE-ABS("abomey-calavi") OR TITLE-ABS("gaborone") OR TITLE-ABS("ouagadougou") OR TITLE-ABS("bobo-dioulasso") OR TITLE-ABS("bujumbura") OR TITLE-ABS("yaounde") OR TITLE-ABS("douala") OR TITLE-ABS("praia") OR TITLE-ABS("bangui") OR TITLE-ABS("n`djamena") OR TITLE-ABS("ndjamena") OR TITLE-ABS("moroni") OR TITLE-ABS("brazzaville") OR TITLE-ABS("pointe-noire") OR TITLE-ABS("kinshasa") OR TITLE-ABS("mbuji-mayi") OR TITLE-ABS("lubumbashi") OR TITLE-ABS("kananga") OR TITLE-ABS("kisangani") OR TITLE-ABS("bukavu") OR TITLE-ABS("yamoussoukro") OR TITLE-ABS("abidjan") OR TITLE-ABS("malabo") OR TITLE-ABS("asmara") OR TITLE-ABS("mbabane") OR TITLE-ABS("addis ababa") OR TITLE-ABS("libreville") OR TITLE-ABS("banjul") OR TITLE-ABS("accra") OR TITLE-ABS("kumasi") OR TITLE-ABS("sekondi takoradi") OR TITLE-ABS("conakry") OR TITLE-ABS("bissau") OR TITLE-ABS("nairobi") OR TITLE-ABS("mombasa") OR TITLE-ABS("mombassa") OR TITLE-ABS("maseru") OR TITLE-ABS("monrovia") OR TITLE-ABS("antananarivo") OR TITLE-ABS("lilongwe") OR TITLE-ABS("blantyre-limbe") OR TITLE-ABS("bamako") OR TITLE-ABS("nouakchott") OR TITLE-ABS("port louis") OR TITLE-ABS("maputo") OR TITLE-ABS("matola") OR TITLE-ABS("nampula") OR TITLE-ABS("windhoek") OR TITLE-ABS("niamey") OR TITLE-ABS("abuja") OR TITLE-ABS("lagos") OR TITLE-ABS("kano") OR TITLE-ABS("ibadan") OR TITLE-ABS("port harcourt") OR TITLE-ABS("kigali") OR TITLE-ABS("sao tome*") OR TITLE-ABS("dakar") OR TITLE-ABS("freetown") OR TITLE-ABS("mogadishu") OR TITLE-ABS("hargeisa") OR TITLE-ABS("hargeysa") OR TITLE-ABS("pretoria") OR TITLE-ABS("cape town") OR TITLE-ABS("johannesburg") OR TITLE-ABS("Soweto") OR TITLE-ABS("durban") OR TITLE-ABS("port elizabeth") OR TITLE-ABS("west rand") OR TITLE-ABS("juba") OR TITLE-ABS("khartoum") OR TITLE-ABS("nyala") OR TITLE-ABS("dar es salaam") OR TITLE-ABS("dodoma") OR TITLE-ABS("mwanza") OR TITLE-ABS("lome") OR TITLE-ABS("kampala") OR TITLE-ABS("lusaka") OR TITLE-ABS("harare"))

AND

(TITLE-ABS("Telehealth") OR TITLE-ABS("Tele-health") OR TITLE-ABS("Telemedic*") OR TITLE-ABS("Tele-medic*") OR TITLE-ABS("telemetry") OR TITLE-ABS("tele-metry") OR TITLE-ABS("biotelemetry") OR TITLE-ABS("radiotelemetry") OR TITLE-ABS("teleradiometry") OR TITLE-ABS("telesensing") OR TITLE-ABS("tele-sensing") OR TITLE-ABS("telemonitor*") OR TITLE-ABS("tele-monitor*") OR TITLE-ABS("telenurs*") OR TITLE-ABS("tele-nurs*") OR TITLE-ABS("teleconsultat*") OR TITLE-ABS("tele-consultat*") OR TITLE-ABS("telerehabilitation*") OR TITLE-ABS("tele-rehabilitation*") OR TITLE-ABS("tele-ophthalmology") OR TITLE-ABS("teleophthalmology") OR ((TITLE-ABS("virtual" OR "remote" OR "distance" OR "distant" OR "e")) W/2 (TITLE-ABS("medic*" OR "monitor*" OR "nurs*" OR "consultat*" OR "rehabilitation*" OR "support*" OR "counsel*" OR "treatment*" OR "therap*" OR "sensing"))) OR TITLE-ABS("emedic*") OR TITLE-ABS("emonitor*") OR TITLE-ABS("enurs*") OR TITLE-ABS("econsultat*") OR TITLE-ABS("erehabilitation") OR TITLE-ABS("esupport*") OR TITLE-ABS("ecounsel*") OR TITLE-ABS("etreatment*") OR TITLE-ABS("etherap*") OR TITLE-ABS("esensing") OR TITLE-ABS("Mhealth") OR TITLE-ABS("M-health") OR TITLE-ABS("mobile health") OR TITLE-ABS("Ehealth") OR TITLE-ABS("E-health") OR TITLE-ABS("e mail*") OR TITLE-ABS("email*") OR ((TITLE-ABS("electronic")) W/2 (TITLE-ABS("mail*" OR "messaging"))) OR TITLE-ABS("hotline*") OR TITLE-ABS("interactive voice response system*") OR ((TITLE-ABS("web" OR "online" OR "on-line" OR "internet" OR "video" OR "virtual" OR "tele")) W/3 (TITLE-ABS("intervention*" OR "conferenc*" OR "communication" OR "seminar*" OR "information*"))) OR TITLE-ABS("internet") OR TITLE-ABS("world wide web") OR TITLE-ABS("www") OR ((TITLE-ABS("mobile" OR "cell" OR "smart" OR "portable")) W/2 (TITLE-ABS("phone*" OR "telephone*"))) OR TITLE-ABS("social media*") OR TITLE-ABS("facebook") OR TITLE-ABS("twitter") OR TITLE-ABS("telecommunication*") OR TITLE-ABS("broadcasting") OR TITLE-ABS("text messag*") OR TITLE-ABS("texting") OR TITLE-ABS("teleconferenc*") OR TITLE-ABS("videoconferenc*") OR TITLE-ABS("webconferenc*") OR TITLE-ABS("webcast*") OR TITLE-ABS("podcast*") OR TITLE-ABS("webinar*") OR TITLE-ABS("SMS") OR TITLE-ABS("telephone*") OR TITLE-ABS("smartphone*") OR ((TITLE-ABS("mobile" OR "portable" OR "tablet")) W/2 (TITLE-ABS("app" OR "apps" OR "application*"))) OR TITLE-ABS("website") OR TITLE-ABS("homepage") OR TITLE-ABS("mobile technology") OR TITLE-ABS("decentrali*") OR ((TITLE-ABS("ambulatory" OR "dispensary" OR "extramural" OR "extra-mural" OR "outpatient" OR "outpatient health*" OR "out-patient" OR "out-patient health*" OR "out-of-office")) W/2 (TITLE-ABS("care" OR "healthcare" OR "service*" OR "setting*" OR "monitoring*" OR "treatment*" OR "therap*"))) OR ((TITLE-ABS("community" OR "community health*" OR "district" OR "public health" OR "nurse-led" OR "pharmacist-led" OR "nurse-based" OR "pharmacist-based" OR "social service" OR "collaborative")) W/3 (TITLE-ABS("care" OR "healthcare" OR "nurs*" OR "integration" OR "service*" OR "program*" OR "medicine" OR "support*" OR "engagement" OR "intervention*" OR "deliver*" OR "outreach" OR "distribution" OR "network*" OR "participatory method*" OR "rehabilitation*" OR "resource*" OR "strateg*" OR "project*" OR "approach*" OR "testing" OR "screening*"))) OR TITLE-ABS("task shar*") OR TITLE-ABS("task shift*") OR ((TITLE-ABS("adult" OR "home" OR "domicil*" OR "domestic" OR "visiting" OR "residential" OR "residence")) W/3 (TITLE-ABS("care") OR ("healthcare") OR ("nurs*") OR ("help") OR ("assistance") OR ("service*") OR ("treatment*") OR ("therap*") OR ("medication review*") OR ("pharmac*") OR ("monitor*") OR ("visit*") OR ("agenc*") OR ("testing") OR ("screening*") OR ("diagnos*"))) OR TITLE-ABS("homecare") OR TITLE-ABS("house call*") OR TITLE-ABS("housebound patient*") OR TITLE-ABS("adult day care") OR TITLE-ABS("family service*") OR TITLE-ABS("family involve*") OR TITLE-ABS("family based") OR TITLE-ABS("family-based") OR TITLE-ABS("family health*") OR TITLE-ABS("out-of-facilit*") OR ((TITLE-ABS("health" OR "health service" OR "sanitary")) W/2 (TITLE-ABS("center*" OR "centre*" OR "clinic*" OR "institute*" OR "unit*" OR "resort*" OR "facility" OR "facilities"))) OR TITLE-ABS("apothecary") OR TITLE-ABS("chemist shop*") OR TITLE-ABS("chemist`s shop*") OR TITLE-ABS("pharmacies") OR TITLE-ABS("pharmacy") OR TITLE-ABS("pharmaceutical service*") OR ((TITLE-ABS("rural" OR "school*" OR "workplace" OR "worksite" OR "work-place" OR "work-site")) W/3 (TITLE-ABS("health*" OR "care" OR "medicine" OR "nurse" OR "nurses" OR "nursing" OR "intervention*" OR "program*" OR "approach*" OR "service*" OR "strateg*"))) OR ((TITLE-ABS("self")) W/2 (TITLE-ABS("care*" OR "help" OR "management" OR "monitoring" OR "treatment*" OR "medication" OR "nurturance" OR "testing" OR "measur*"))) OR TITLE-ABS("selfcare") OR TITLE-ABS("selfhelp") OR TITLE-ABS("selfmanagement") OR TITLE-ABS("selfmonitor*") OR TITLE-ABS("selftreatment") OR TITLE-ABS("selfmedication*") OR ((TITLE-ABS("social" OR "spiritual")) W/2 (TITLE-ABS("care" OR "support" OR "network*" OR "service*" OR "work"))) OR TITLE-ABS("support group*") OR TITLE-ABS("support program*") OR TITLE-ABS("paramedical") OR TITLE-ABS("para-medical") OR TITLE-ABS("non-health") OR TITLE-ABS("non-healthcare") OR TITLE-ABS("non-medical") OR ((TITLE-ABS("health" OR "healthcare")) W/2 (TITLE-ABS("support worker*" OR "assistant*" OR "aid*"))) OR ((TITLE-ABS("community" OR "district" OR "public health" OR "assistan*" OR "aid*" OR "staff*" OR "manpower" OR "personnel")) W/4 (TITLE-ABS("nurse" OR "nurses" OR "nursing" OR "matron*"))) OR TITLE-ABS("midlevel health professional*") OR TITLE-ABS("midlevel health provider*") OR ((TITLE-ABS("community" OR "community based" OR "village" OR "lay" OR "allied health" OR "auxiliary health" OR "health auxiliary")) W/3 (TITLE-ABS("worker*" OR "provider*" OR "personnel" OR "profession*" OR "staff" OR "aide*" OR "supporter*" OR "volunteer*" OR "advocate*" OR "team*" OR "group*" OR "pharmacist*"))) OR TITLE-ABS("barefoot doctor*") OR TITLE-ABS("local supervisor*") OR TITLE-ABS("caregiver*") OR TITLE-ABS("care giver*") OR ((TITLE-ABS("peer")) W/2 (TITLE-ABS("group*" OR "support" OR "relation*" OR "education"))) OR TITLE-ABS("participatory research") OR TITLE-ABS("patient involvement") OR TITLE-ABS("patient participation") OR TITLE-ABS("patient empowerment") OR TITLE-ABS("patient activation") OR TITLE-ABS("patient engagement"))

| **Section and Topic** | **Item #** | **Checklist item** | **Location where item is reported** |
| --- | --- | --- | --- |
| **TITLE** | | |  |
| Title | 1 | Identify the report as a systematic review. | Page 1 |
| **ABSTRACT** | | |  |
| Abstract | 2 | See the PRISMA 2020 for Abstracts checklist. | Page 1 |
| **INTRODUCTION** | | |  |
| Rationale | 3 | Describe the rationale for the review in the context of existing knowledge. | Page 2 |
| Objectives | 4 | Provide an explicit statement of the objective(s) or question(s) the review addresses. | Page 3 |
| **METHODS** | | |  |
| Eligibility criteria | 5 | Specify the inclusion and exclusion criteria for the review and how studies were grouped for the syntheses. | Page 3 |
| Information sources | 6 | Specify all databases, registers, websites, organisations, reference lists and other sources searched or consulted to identify studies. Specify the date when each source was last searched or consulted. | Page 5 |
| Search strategy | 7 | Present the full search strategies for all databases, registers and websites, including any filters and limits used. | Page 5 |
| Selection process | 8 | Specify the methods used to decide whether a study met the inclusion criteria of the review, including how many reviewers screened each record and each report retrieved, whether they worked independently, and if applicable, details of automation tools used in the process. | Page 5 |
| Data collection process | 9 | Specify the methods used to collect data from reports, including how many reviewers collected data from each report, whether they worked independently, any processes for obtaining or confirming data from study investigators, and if applicable, details of automation tools used in the process. | Page 6 |
| Data items | 10a | List and define all outcomes for which data were sought. Specify whether all results that were compatible with each outcome domain in each study were sought (e.g. for all measures, time points, analyses), and if not, the methods used to decide which results to collect. | Page 5 |
|  | 10b | List and define all other variables for which data were sought (e.g. participant and intervention characteristics, funding sources). Describe any assumptions made about any missing or unclear information. | Page 5 |
| Study risk of bias assessment | 11 | Specify the methods used to assess risk of bias in the included studies, including details of the tool(s) used, how many reviewers assessed each study and whether they worked independently, and if applicable, details of automation tools used in the process. | Page 6 and 7 |
| Effect measures | 12 | Specify for each outcome the effect measure(s) (e.g. risk ratio, mean difference) used in the synthesis or presentation of results. | Not available as Scoping review |
| Synthesis methods | 13a | Describe the processes used to decide which studies were eligible for each synthesis (e.g. tabulating the study intervention characteristics and comparing against the planned groups for each synthesis (item #5)). | Page 4 |
|  | 13b | Describe any methods required to prepare the data for presentation or synthesis, such as handling of missing summary statistics, or data conversions. | Not available, as no metaanalisis |
|  | 13c | Describe any methods used to tabulate or visually display results of individual studies and syntheses. | Page 5 |
|  | 13d | Describe any methods used to synthesize results and provide a rationale for the choice(s). If meta-analysis was performed, describe the model(s), method(s) to identify the presence and extent of statistical heterogeneity, and software package(s) used. | Page 5 |
|  | 13e | Describe any methods used to explore possible causes of heterogeneity among study results (e.g. subgroup analysis, meta-regression). | Not available, as no metaanalisis |
|  | 13f | Describe any sensitivity analyses conducted to assess robustness of the synthesized results. | Not available, as no metaanalisis |
| Reporting bias assessment | 14 | Describe any methods used to assess risk of bias due to missing results in a synthesis (arising from reporting biases). | Not available, as no metaanalisis |
| Certainty assessment | 15 | Describe any methods used to assess certainty (or confidence) in the body of evidence for an outcome. | Page 5 |
| **RESULTS** | | |  |
| Study selection | 16a | Describe the results of the search and selection process, from the number of records identified in the search to the number of studies included in the review, ideally using a flow diagram. | Page 6 |
|  | 16b | Cite studies that might appear to meet the inclusion criteria, but which were excluded, and explain why they were excluded. | Page 6 |
| Study characteristics | 17 | Cite each included study and present its characteristics. | Page 7 and tables |
| Risk of bias in studies | 18 | Present assessments of risk of bias for each included study. | Page 21 and table |
| Results of individual studies | 19 | For all outcomes, present, for each study: (a) summary statistics for each group (where appropriate) and (b) an effect estimate and its precision (e.g. confidence/credible interval), ideally using structured tables or plots. | Not available, as no metaanalisis |
| Results of syntheses | 20a | For each synthesis, briefly summarise the characteristics and risk of bias among contributing studies. | Not available, as no metaanalisis |
|  | 20b | Present results of all statistical syntheses conducted. If meta-analysis was done, present for each the summary estimate and its precision (e.g. confidence/credible interval) and measures of statistical heterogeneity. If comparing groups, describe the direction of the effect. | Not available, as no metaanalisis |
|  | 20c | Present results of all investigations of possible causes of heterogeneity among study results. | Not available, as no metaanalisis |
|  | 20d | Present results of all sensitivity analyses conducted to assess the robustness of the synthesized results. | Not available, as no metaanalisis |
| Reporting biases | 21 | Present assessments of risk of bias due to missing results (arising from reporting biases) for each synthesis assessed. | Not available, as no metaanalisis |
| Certainty of evidence | 22 | Present assessments of certainty (or confidence) in the body of evidence for each outcome assessed. | Page 21 and table |
| **DISCUSSION** | | |  |
| Discussion | 23a | Provide a general interpretation of the results in the context of other evidence. | Page 7 and 8 |
|  | 23b | Discuss any limitations of the evidence included in the review. | Page 23 |
|  | 23c | Discuss any limitations of the review processes used. | Page 23 |
|  | 23d | Discuss implications of the results for practice, policy, and future research. | Page 24 |
| **OTHER INFORMATION** | | |  |
| Registration and protocol | 24a | Provide registration information for the review, including register name and registration number, or state that the review was not registered. | Not register as scoping review |
|  | 24b | Indicate where the review protocol can be accessed, or state that a protocol was not prepared. | Page 3 |
|  | 24c | Describe and explain any amendments to information provided at registration or in the protocol. | No applicable |
| Support | 25 | Describe sources of financial or non-financial support for the review, and the role of the funders or sponsors in the review. | Page 25 |
| Competing interests | 26 | Declare any competing interests of review authors. | Page 25 |
| Availability of data, code and other materials | 27 | Report which of the following are publicly available and where they can be found: template data collection forms; data extracted from included studies; data used for all analyses; analytic code; any other materials used in the review. | Literature search string and used tools available in extended material file |

*From:*  Page MJ, McKenzie JE, Bossuyt PM, Boutron I, Hoffmann TC, Mulrow CD, et al. The PRISMA 2020 statement: an updated guideline for reporting systematic reviews. BMJ 2021;372:n71. doi: 10.1136/bmj.n71

For more information, visit: <http://www.prisma-statement.org/>
